# Prediction of Major Adverse Cardiovascular Events in Peripheral Artery Disease: Integrating Metabolomics and Proteomics for Risk Stratification

**DOI:** 10.34133/research.1229

**Published:** 2026-05-06

**Authors:** Wenxin Zhao, Lingbing Meng, Sheng Yan, Peng Li, Youjing Sun, Yaming Guo, Bowen Zhang, Yifan Cao, VIP-PADS Study Collaborators, Junhong Ren, Yongjun Li, Zuoguan Chen

**Affiliations:** ^1^ Department of Vascular Surgery, Beijing Hospital, National Center for Gerontology; National Clinical Research Center for Gerontology; The Key Laboratory of Geriatrics of NHC; Institute of Geriatric Medicine, Chinese Academy of Medical Sciences, P.R. China.; ^2^ Beijing Hospital, National Center of Gerontology, Institute of Geriatric Medicine, Chinese Academy of Medical Sciences & Peking Union Medical College, Beijing, P.R. China.; ^3^ Cardiometabolic Medicine Center, National Clinical Research Center for Cardiovascular Diseases, Fuwai Hospital, National Center for Cardiovascular Diseases, Chinese Academy of Medical Sciences and Peking Union Medical College, Beijing 100037, P.R. China.; ^4^ Translational Medical Center, Weifang Second People’s Hospital, Weifang, P.R. China.; ^5^ Department of Vascular Surgery, Second Hospital of Shanxi Medical University, Taiyuan 030000, P.R. China.; ^6^ The Key Laboratory of Geriatrics, Beijing Institute of Geriatrics, Institute of Geriatric Medicine, Chinese Academy of Medical Sciences, Beijing Hospital/National Center of Gerontology of National Health Commission, Beijing, P.R. China.; ^7^ Department of Sonography, Beijing Hospital, National Center for Gerontology, National Clinical Research Center for Gerontology, The Key Laboratory of Geriatrics of NHC, Institute of Geriatric Medicine, Chinese Academy of Medical Sciences, P.R. China

## Abstract

Peripheral artery disease (PAD) confers elevated risk for major adverse cardiovascular events (MACE), yet accurate risk stratification remains a challenge, particularly among patients with advanced disease necessitating endovascular revascularization. This study aimed to improve the prediction of MACE in a clearly defined high-risk PAD population (hospitalized patients undergoing endovascular intervention) by identifying novel protein biomarkers and developing a robust risk model. We prospectively analyzed blood samples from 164 hospitalized PAD patients scheduled for endovascular revascularization, employing untargeted plasma proteomics and metabolomics. Differential protein and metabolite profiles were compared between patients with and without subsequent MACE. Several proteins, including MMP3, MMP19, and PRB2, were markedly elevated in patients who developed MACE. A proteomics-based risk model incorporating these biomarkers achieved high discriminative accuracy (area under the curve > 0.80) for identifying individuals at increased risk. Metabolomic profiling revealed additional pathway alterations, notably involving tryptophan and glycogen metabolism, which provided mechanistic insights into cardiovascular complications but were not directly incorporated into the prediction model. This study demonstrates that integrating protein biomarkers markedly improves risk stratification in advanced PAD patients undergoing surgical intervention. The findings offer promising tools for early detection and enable more personalized management for this high-risk subgroup, while also deepening understanding of disease pathophysiology. However, further validation in larger and more diverse prospective cohorts is warranted before these findings can be broadly applied in clinical practice.

## Introduction

Peripheral artery disease (PAD), characterized by arterial occlusion in the lower extremities, affects over 230 million individuals worldwide [1]. PAD is closely associated with an increased risk of major adverse cardiovascular events (MACE), including myocardial infarction, stroke, and cardiovascular death, and poses a substantial social and economic burden [2–4]. In advanced PAD, patients commonly exhibit symptoms such as lifestyle-limiting claudication, critical limb ischemia, or complex comorbidities. For these individuals, endovascular revascularization is often the preferred strategy to restore limb perfusion and function. Importantly, patients who are hospitalized for such endovascular interventions typically present with more advanced disease (predominantly Fontaine stages IIb to IV) and a higher prevalence of severe comorbidities. Precisely because of this severe disease burden and multiple comorbidities, these patients face an increased risk of both perioperative and longer-term MACE [5]. Therefore, there is an urgent need for improved risk stratification and individualized management in this high-risk subgroup.

Despite advances in therapeutic interventions, early detection of PAD and effective risk stratification for subsequent MACE remain significant clinical challenges, primarily due to the lack of reliable and robust biomarkers. Traditional risk factors and commonly used assessment tools, such as age, smoking, diabetes, and ankle–brachial index, are associated with increased cardiovascular risk but lack the sensitivity and specificity required to accurately identify PAD patients at highest risk for MACE [5,6]. This limitation is particularly evident among surgical inpatients with advanced disease, where precise risk assessment is crucial for perioperative management. Accordingly, there is a pressing need for novel, sensitive, and specific biomarkers to inform personalized prognosis and optimize perioperative surveillance in this population. In response to this unmet clinical need, recent research has increasingly focused on the identification of novel biomarkers, especially those related to inflammatory pathways, to improve cardiovascular risk assessment in PAD [7].

Recent advances in omics technologies, such as proteomics and metabolomics, have greatly enhanced our understanding of the molecular mechanisms underlying cardiovascular diseases [8]. Proteomics enables the comprehensive profiling and quantification of protein expression and function, while metabolomics provides insights into small-molecule dynamics and cellular metabolic pathways. Taken together, the integration of these multiomics modalities offers a promising strategy to identify novel biomarkers, elucidate disease mechanisms, and improve risk prediction in PAD [9].

To address this gap, we conducted a study exclusively among a prospective cohort of hospitalized PAD patients scheduled for endovascular revascularization. By targeting this clearly defined high-risk cohort, we applied untargeted plasma proteomic and metabolomic analyses to identify molecular alterations associated with MACE and to develop an integrated, protein-based risk prediction model. This approach aims to advance precision cardiovascular medicine by enabling more accurate risk stratification and individualized management for advanced PAD patients undergoing endovascular intervention [10].

## Results

### Clinical characteristics of the studied cohort

**Fig. 1.**
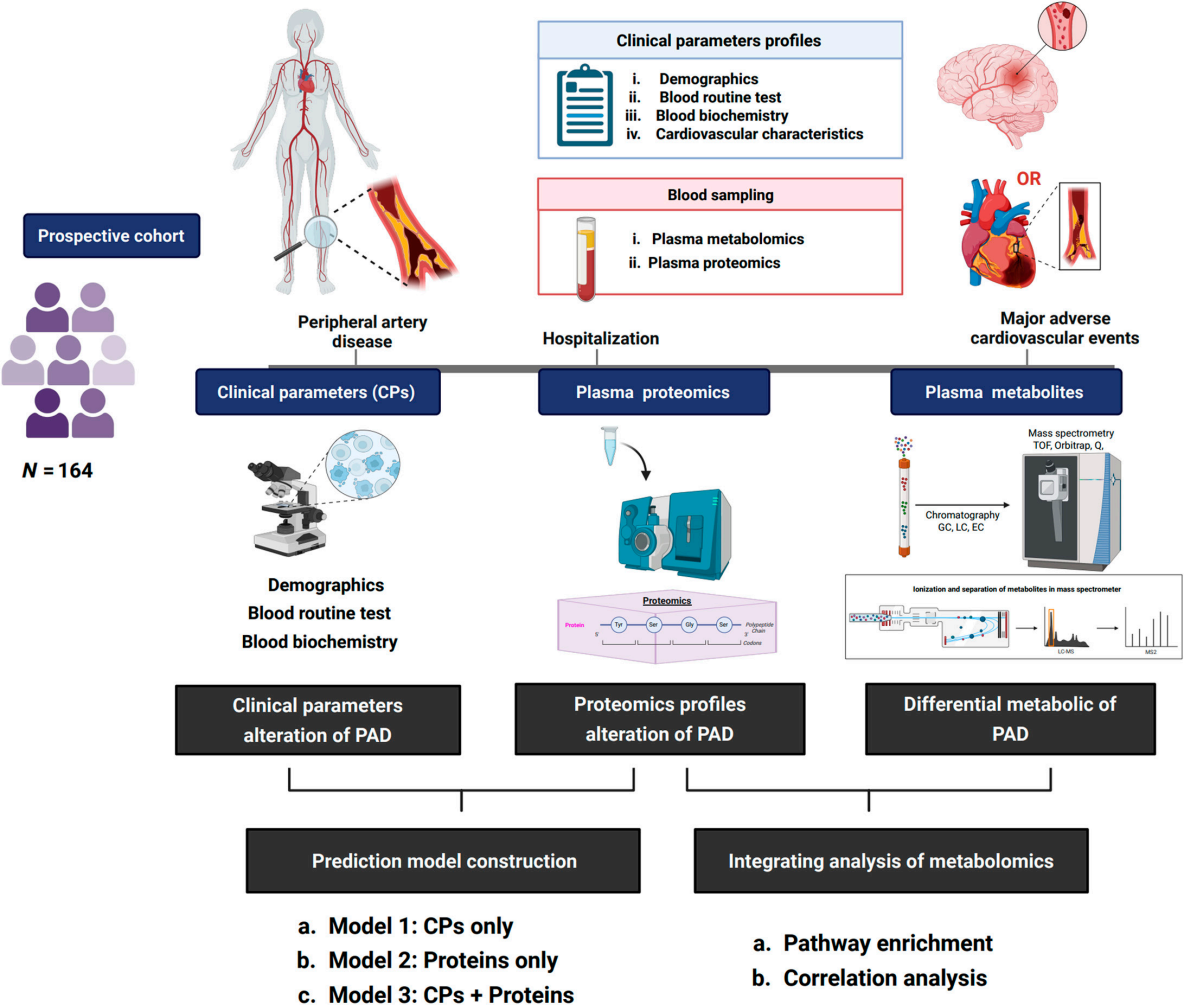
Study design and multiomics workflow for risk prediction in PAD. This figure illustrates the framework of a prospective cohort study enrolling 164 patients with PAD. At hospitalization, clinical parameters including demographics, routine blood tests, biochemistry, and cardiovascular characteristics, as well as blood samples for plasma proteomic and metabolomic profiling, were collected. All patients were prospectively followed for MACE. Clinical, proteomic, and metabolomic data were analyzed to explore disease mechanisms and build risk prediction models. CP, clinical parameters; PAD, peripheral artery disease; MACE, major adverse cardiovascular events.

Figure 1 depicts the overall design and analytical workflow of this study. A total of 175 patients with PAD were initially screened and prospectively recruited according to strict inclusion and exclusion criteria. Of these, 11 patients were lost to follow-up or withdrew consent, leaving 164 patients included in the final analysis. The detailed patient recruitment and follow-up flowchart is provided in Fig. S1. During the hospitalization period, baseline blood samples were collected for plasma proteomics and metabolomics analysis, and comprehensive clinical parameters including demographic characteristics, routine laboratory tests, biochemical indicators, comorbidities, and cardiovascular features were recorded. All blood samples for omics analysis were collected immediately after admission and prior to the initiation of any in-hospital, peri-procedural intravenous or high-dose antithrombotic therapy. Baseline medications (as recorded in Table 1) were defined as chronic oral drugs taken at home before hospitalization and before any intervention. All patients underwent longitudinal follow-up at prespecified intervals to monitor the occurrence of MACE, which were adjudicated according to standardized definitions (including cardiovascular death, myocardial infarction, and stroke), and confirmed via medical records and telephone interviews. During the follow-up period, 29 patients experienced MACE events, while the remaining 135 patients did not have any related events.

**Table 1.** Clinical features of the patients in the prospective cohort. Data are shown as *N* (%), mean ± SD, or median (IQR), where *N* is the total number of patients with available data. Baseline medication status refers to chronic drugs taken prior to hospitalization; all peri-procedural antithrombotic therapy was initiated after blood sampling.

Characteristic	Missing values	Overall (*N* = 164)	Non-MACE (*N* = 135)	MACE (*N* = 29)	*P* value
Demographic character					
Age	0 (0%)	69.32 ± 8.85	69.07 ± 9.04	70.52 ± 7.94	0.389
Male sex	0 (0%)	127 (77)	103 (76)	24 (83)	0.61
BMI	1 (0.61%)	23.92 ± 3.12	24.01 ± 3.19	23.5 ± 2.83	0.397
Comorbidities					
Smoking	0 (0%)	65 (40)	53 (39)	12 (41)	0.998
Hypertension	0 (0%)	115 (70)	89 (66)	26 (90)	0.021
Diabetes mellitus	0 (0%)	94 (57)	78 (58)	16 (55)	0.96
Hyperlipidemia	0 (0%)	73 (45)	62 (46)	11 (38)	0.562
Chronic kidney disease	0 (0%)	13 (8)	7 (5)	6 (21)	0.013
COPD	0 (0%)	1 (1)	1 (1)	0 (0)	1
Coronary heart disease	0 (0%)	57 (35)	43 (32)	14 (48)	0.141
Atrial fibrillation	0 (0%)	6 (4)	5 (4)	1 (3)	1
History of CABG	0 (0%)	3 (2)	2 (1)	1 (3)	0.444
History of PCI	0 (0%)	12 (7)	8 (6)	4 (14)	0.228
Cerebrovascular disease	0 (0%)	48 (29)	36 (27)	12 (41)	0.175
Carotid artery stenosis	0 (0%)	22 (13)	18 (13)	4 (14)	1
Renal artery stenosis	0 (0%)	1 (1)	1 (1)	0 (0)	1
Malignancy	0 (0%)	12 (7)	7 (5)	5 (17)	0.039
Fontaine	0 (0%)				0.375
II		87 (53)	74 (55)	13 (45)	
III		22 (13)	16 (12)	6 (21)	
IV		55 (34)	45 (33)	10 (34)	
Preadmission chronic medications					
Anticoagulants drugs	0 (0%)	144 (88)	118 (87)	26 (90)	1
Antihypertensive drugs	0 (0%)	109 (66)	86 (64)	23 (79)	0.162
Lipid-lowering drugs	0 (0%)	124 (76)	101 (75)	23 (79)	0.785
Vasodilator drugs	0 (0%)	48 (29)	41 (30)	7 (24)	0.657
Laboratory measurements					
White blood cell count (×10^9^/l)	2 (1.22%)	7 (5.41, 7.86)	6.96 (5.39, 7.84)	7.07 (5.85, 8.26)	0.663
Red blood cell count (×10^12^/l)	3 (1.83%)	4.11 ± 0.62	4.18 ± 0.58	3.82 ± 0.74	0.018
Hemoglobin concentration (g/dl)	2 (1.22%)	129 (113, 141)	130 (115, 141.5)	113 (103, 135)	0.014
Neutrophil count (×10^9^/l)	2 (1.22%)	4.36 (3.4, 5.59)	4.28 (3.29, 5.5)	4.74 (3.88, 6.19)	0.465
Lymphocyte count	2 (1.22%)	1.62 (1.23, 2.11)	1.65 (1.27, 2.19)	1.35 (0.9, 1.74)	0.022
Platelet count	3 (1.83%)	217.5 (172.75, 251.25)	219 (174, 260.5)	200 (166, 240)	0.271
Aspartate aminotransferase (U/l)	1 (0.61%)	17 (13, 20)	17 (13.5, 20)	17 (11, 24)	0.837
Alanine aminotransferase (U/l)	1 (0.61%)	16 (11, 23)	16 (11.5, 22.5)	16 (9, 25)	0.57
Creatinine (μmol/l)	1 (0.61%)	76 (63, 93)	74 (62, 86.5)	83 (74, 161)	0.002
Urea	4 (2.44%)	5.97 (4.95, 7.69)	5.88 (4.86, 7.11)	7.52 (5.64, 10.3)	0.011
Blood glucose	12 (7.32%)	5.8 (5, 7.23)	5.8 (5.05, 7.2)	5.7 (4.7, 7.3)	0.719
Albumin (g/l)	11 (6.71%)	37 (35, 40)	37.55 (36, 40)	36 (33, 38)	0.005
BNP	25 (15.24%)	53.01 (28, 123.65)	50.15 (26.87, 107.99)	123.66 (44.95, 385.28)	0.02
Outcome					
Follow-up time	0 (0%)	15.27 (8.53, 22.58)	16 (9.48, 23.25)	10.4 (4.1, 17.1)	0.006

MACE, major adverse cardiovascular events; BMI, body mass index; COPD, chronic obstructive pulmonary disease; CABG, coronary artery bypass grafting; PCI, percutaneous coronary intervention; BNP, B-type natriuretic peptide.

Baseline comparison between patients with and without MACE is summarized in Table 1. There were no statistically significant differences in age, sex, body mass index, or smoking status between groups. However, a higher prevalence of hypertension (90% vs. 66%, *P* = 0.021), chronic kidney disease (21% vs. 5%, *P* = 0.013), and malignancy (17% vs. 5%, *P* = 0.039) was observed among MACE cases, suggesting a potential association with cardiovascular risk. Chronic anticoagulant therapy (preadmission) was reported in 88% of patients. All peri-procedural antithrombotic agents were started only after blood sampling. Laboratory indices also differed: the MACE group had lower hemoglobin (113 vs. 130 g/dl, *P* = 0.014), lower lymphocyte count (1.35 vs. 1.65 × 10^9^/l, *P* = 0.022), higher creatinine (83 vs. 74 μmol/l, *P* = 0.002), higher urea, lower albumin, and notably elevated BNP levels (123.66 vs. 50.15, *P* = 0.02). Median follow-up time was shorter among MACE patients (10.4 vs. 16 months, *P* = 0.006). A total of 29 MACE events occurred during the follow-up period; of these, 4 events occurred within the first 90 days after intervention. These findings are observed as important clinical correlates of poor cardiovascular outcomes in PAD.

### Differential proteins profiles

**Fig. 2.**
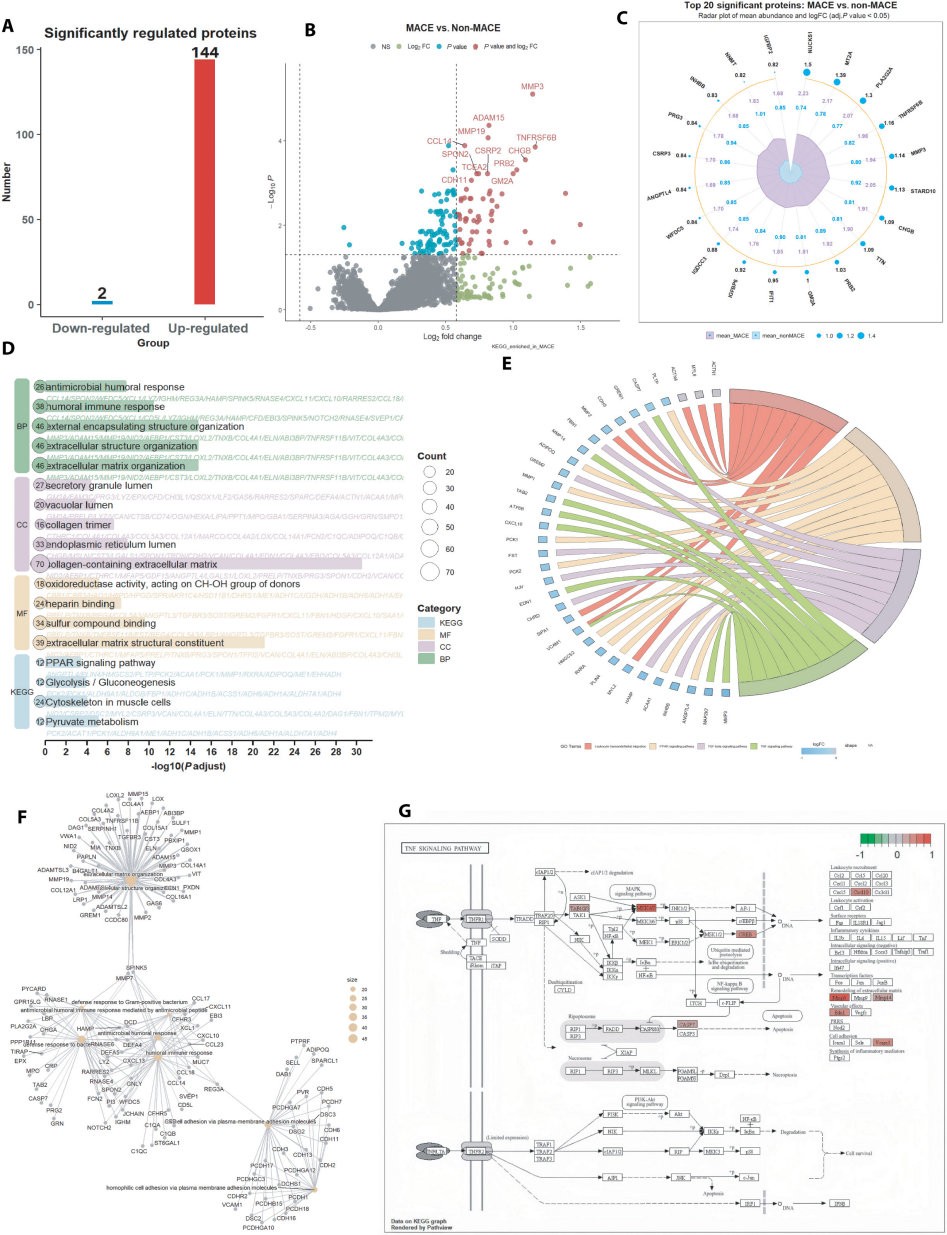
Proteomics differential analysis reveals proteomic alterations and pathway enrichment underlying major adverse cardiovascular events (MACE) in PAD patients. (A) Bar plot of significantly regulated proteins (adjusted *P* < 0.05) identified by proteomics differential analysis. A total of 144 proteins were up-regulated and 2 were down-regulated in MACE versus non-MACE patients. (B) Volcano plot of proteomic changes between MACE and non-MACE, highlighting especially up-regulated proteins with |log2 fold change| > 0.58 and adjusted *P* value < 0.05. Key inflammatory and matrix-remodeling factors are annotated. (C) Radar plot underscores the magnitude of dysregulation in these effector proteins, supporting their roles as potential biomarkers and therapeutic targets. (D) Functional enrichment analysis of differentially expressed proteins, with GO/KEGG terms covering humoral immunity, extracellular matrix, and metabolic processes. (E) Chord diagram mapping enriched KEGG pathways (*P* value < 0.05) to their contributing proteins, showing areas of immune activation and remodeling. (F) Protein interaction network of KEGG-enriched proteins, presenting functional connectivity and hubs of disease biology. (G) TNF-α signaling pathway map, highlighting up-regulated proteins in MACE patients within the pathway, suggesting a mechanistic link to vascular inflammation and adverse outcomes. MACE, major adverse cardiovascular events; GO, Gene Ontology; KEGG, Kyoto Encyclopedia of Genes and Genomes; BP, biological process; CC, cellular component; MF, molecular function; PPI, protein–protein interaction; TNF, tumor necrosis factor.

To elucidate the molecular mechanisms underlying MACE in PAD patients, we performed comprehensive differential proteomic profiling. In total, 146 plasma proteins were significantly dysregulated between patients with and without MACE (adjusted *P* < 0.05; Fig. 2A and Table S1), of which 144 were up-regulated and only 2 were down-regulated, suggesting widespread activation of pathological pathways in high-risk individuals. No evidence of major batch effects or technical drift was observed based on standard quality control analyses (Figs. S2 to S4). The volcano plot (Fig. 2B and Data S1) shows that the up-regulated proteins predominate in the MACE group. The 20 proteins (Table S1) with the most significant differential expression include mediators of vascular remodeling and inflammation (MMP3 and MMP19), immune and extracellular matrix (ECM)-related factors (ADAM15, CCL14, and TNFRSF6B), and regulators of lipid metabolism and atherosclerosis (AEBP1 and GM2A). The radar plot (Fig. 2C) further underscores the persistent elevation of these proteins in the MACE group. Besides canonical matrix metalloproteinases and immune receptors, marked up-regulation of proteins linked to cellular stress responses (NUCKS1), growth factor signaling (IGFBP2 AND IGFBP6), and metabolic regulation (STARD10) highlights the complex interplay of inflammatory, metabolic, and structural remodeling processes in PAD-related MACE. Pathway enrichment analysis (Fig. 2D and Data S2 and S3) revealed pronounced overrepresentation of humoral immune response, ECM organization, and antimicrobial humoral defense, with strong Kyoto Encyclopedia of Genes and Genomes (KEGG) enrichment in peroxisome proliferator-activated receptor (PPAR) signaling, pyruvate metabolism, glycolysis/gluconeogenesis, and multiple immune-related cascades. The recurrent identification of ECM-related proteins (MMPs and COL family) and humoral immune factors further supports the centrality of matrix remodeling and immune dysregulation in MACE pathogenesis.

To further elucidate protein–pathway interactions, a chord diagram (Fig. 2E) revealed extensive cross-talk among up-regulated proteins and key biological processes. Notably, proteins involved in innate/adaptive immunity (CCL14 and CHGB), tissue remodeling (MMP3, MMP19, ADAM15, SPON2, and CSRP2), and metabolic regulation (STARD10 and IGFBP family) were frequently up-regulated, indicating an integrated inflammatory, vascular structural, and metabolic pathophysiology underlying adverse outcomes. Network mapping (Fig. 2F) identified several hub proteins, including matrix metalloproteinases and tumor necrosis factor (TNF) receptor superfamily members, bridging immune with remodeling pathways and reinforcing the multifactorial mechanism of MACE. Given the repeated enrichment of inflammatory and immune-related processes, targeted analysis of TNF-α signaling (Fig. 2G) revealed consistent up-regulation of key proteins involved in matrix metabolism and cell fate regulation, highlighting excessive TNF-α signaling as a central driver of persistent immune activation, vascular pathologic remodeling, and adverse cardiovascular outcomes in PAD. Collectively, these findings emphasize pervasive molecular reprogramming in high-risk individuals, characterized by ECM disruption, metabolic dysregulation, and aberrant immune signaling, which collectively contribute to the development of MACE.

To evaluate the robustness of these findings, we performed sensitivity analyses with sequential covariate adjustments. When preadmission antithrombotic medication use was included as a covariate (Table S2), and when major baseline comorbidities (chronic kidney disease, malignancy, and hypertension) were included as covariates (Table S3), most differentially expressed proteins remained consistent with the primary analysis, with only a small subset exhibiting notable changes in effect size or statistical significance. These results indicate that the observed proteomic differences are robust to potential confounding by either medication exposure or comorbidity burden. A comparative summary of these analyses is provided in Data S4 and S5.

### Differential metabolite profiles

**Fig. 3.**
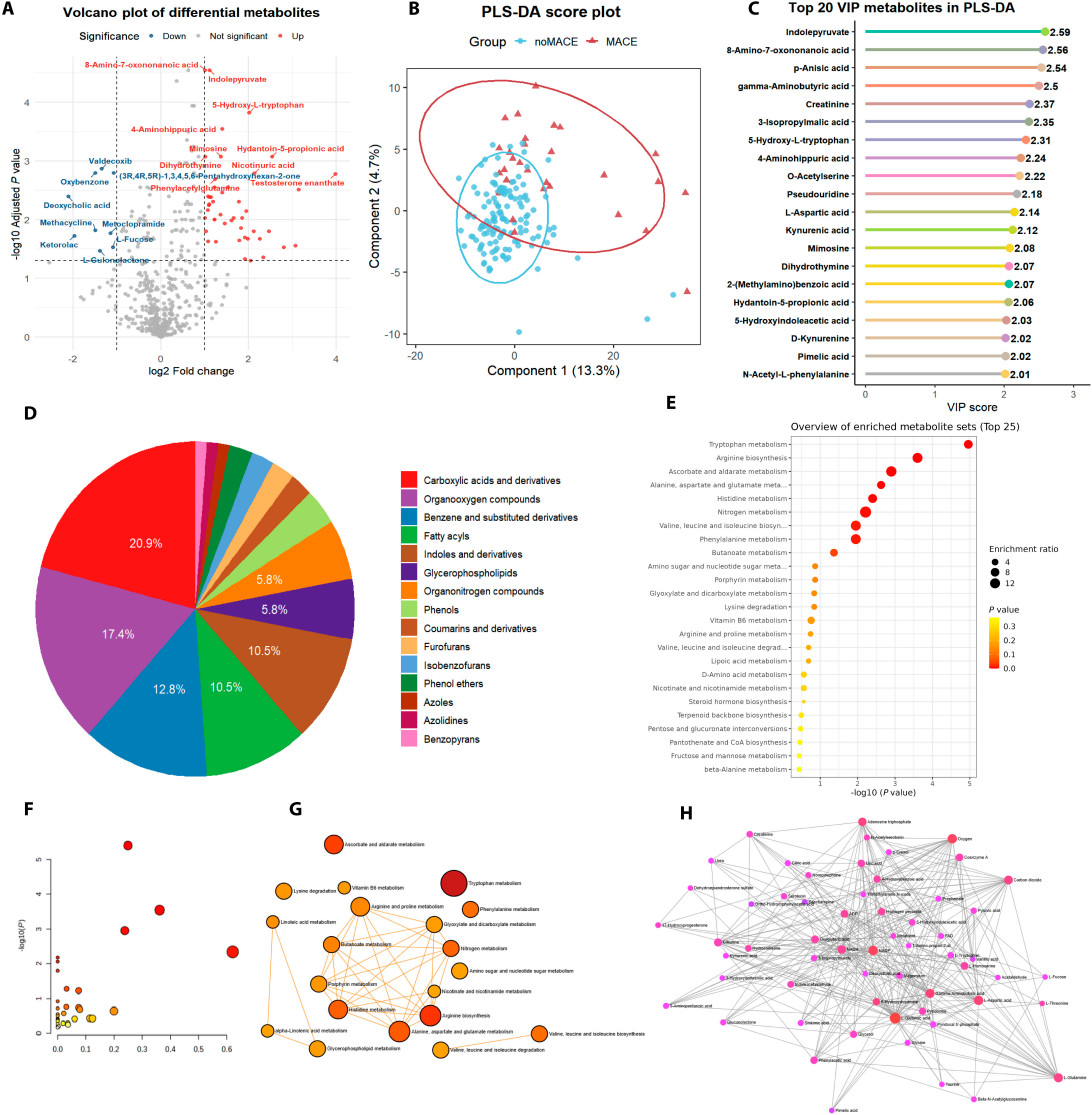
Differential metabolomic profiles and pathway enrichment analysis between PAD patients with and without MACE. (A) Volcano plot of differential metabolites: Shows significantly up-regulated and down-regulated metabolites between MACE and non-MACE groups (adjusted *P* < 0.05, |log2FC| > 1). (B) PLS-DA score plot: Depicts clear separation of MACE and non-MACE groups based on their global metabolic profiles. (C) Top 20 VIP metabolites in PLS-DA: Lists the most discriminatory metabolites driving class separation. (D) Pie chart of metabolite classes: Displays the proportion of major compound classes among significant metabolites, with carboxylic acids and derivatives most prevalent. (E) Dot plot of KEGG pathway enrichment, displaying enrichment ratios and statistical significance for major metabolic pathways. (F) Bubble plot of pathway enrichment results, visualizing enrichment significance and metabolite involvement across pathways. (G) Pathway interaction network: Illustrates connectivity among enriched metabolic pathways, with tryptophan metabolism at the core of multiple metabolic disturbances. (H) Metabolite–metabolite interaction network of significant metabolites. Nodes represent metabolites, and edges indicate significant pairwise associations. PLS-DA, partial least squares discriminant analysis; VIP, variable importance in projection; MACE, major adverse cardiovascular events; noMACE, without major adverse cardiovascular events; FDR, false discovery rate (if applied); enrichment ratio, the observed-to-expected ratio of metabolites in a pathway.

Comprehensive metabolomic profiling was employed to assess long-term prognostic differences in PAD patients. As shown in Fig. 3A (Data S6), volcano plot analysis using limma (adjusted *P* < 0.05, |log2FC| > 1) revealed a distinct set of dysregulated metabolites. Notably, most significant metabolites were up-regulated in the MACE group, such as indolepyruvate, 8-amino-7-oxononanoic acid, 4-aminohippuric acid, and 5-hydroxy-L-tryptophan, which are implicated in amino acid and nitrogen metabolism. A detailed list of the top 30 up-regulated metabolites, along with their log fold change (logFC) and adjusted *P* values, is provided in Table S4. In contrast, metabolites including valdecoxib, oxybenzone, deoxycholic acid, and L-gulonolactone were markedly down-regulated, suggesting disruption of xenobiotic, bile acid, and antioxidative pathways. Multivariate modeling using partial least squares–discriminant analysis (PLS-DA) (Fig. 3B) demonstrated clear stratification of MACE versus non-MACE samples, supporting a metabolic reprogramming landscape associated with adverse cardiovascular outcomes. PLS-DA was used for supervised exploratory visualization. To reduce overfitting risk, model robustness was assessed by cross-validation and permutation testing (Supplementary Methods and Figs. S5 to S7).

The variable importance in projection (VIP) plot (Fig. 3C) identified the top 20 metabolites contributing most heavily to the group separation, with indolepyruvate, 8-amino-7-oxononanoic acid, p-anisic acid, gamma-aminobutyric acid, creatinine, and 3-isopropylmalic acid showing the highest VIP scores. These key metabolites are primarily involved in amino acid metabolism, xenobiotic processing, and energy homeostasis. Class distribution analysis of differential metabolites (Fig. 3D) showed that carboxylic acids and derivatives accounted for the largest proportion (20.9%), followed by organooxygen compounds (17.4%), benzene and substituted derivatives (12.8%), and fatty acyls (10.5%). This indicates an imbalance in central metabolic networks, including organic acid metabolism, fatty acid turnover, and aromatic compound biosynthesis in MACE subjects.

Pathway enrichment analysis using KEGG revealed significant alterations in several metabolic routes (Fig. 3E and F and Data S7). Tryptophan metabolism; arginine biosynthesis; ascorbate and aldarate metabolism; alanine, aspartate, and glutamate metabolism; and histidine metabolism were among the top enriched pathways. These findings highlighted disruptions in amino acid utilization, nucleotide balance, redox regulation, and energy metabolism. Network visualization of enriched metabolic pathways (Fig. 3G) demonstrated that tryptophan metabolism and arginine biosynthesis are at the intersection of multiple perturbed networks, acting as central hubs for broader metabolic disturbances in the MACE cohort. Finally, metabolite interaction mapping (Fig. 3H) revealed a highly interconnected web of up-regulated metabolites, with hub nodes such as glutamine, creatinine, citric acid, serotonin, and NAD, which are crucial for energy metabolism, neurotransmission, and cell signaling. These extensive interactions underscore the complexity of metabolic reprogramming in patients experiencing poor cardiovascular outcomes. These network visualizations are descriptive and hypothesis-generating and do not establish direct biochemical regulation or causality.

To assess robustness, sensitivity analyses adjusting for baseline antithrombotic therapy (Table S5) and major comorbidities (Table S6) were performed. As shown in Table S4, the overall pattern and statistical significance of dysregulated metabolites remained consistent with the primary (unadjusted) results, with only minor changes observed in a small subset of metabolites. These results indicate that the observed metabolic alterations are robust and not substantially confounded by baseline medication use. Additionally, when major baseline comorbidities (chronic kidney disease, malignancy, and hypertension) were included as covariates, the core set of differentially expressed metabolites remained largely unchanged, further supporting that these metabolic signatures are associated primarily with MACE risk rather than comorbidity burden or medication exposure. A comparative summary of these analyses is provided in Data S8 and S9.

### Multiomics integration

**Fig. 4.**
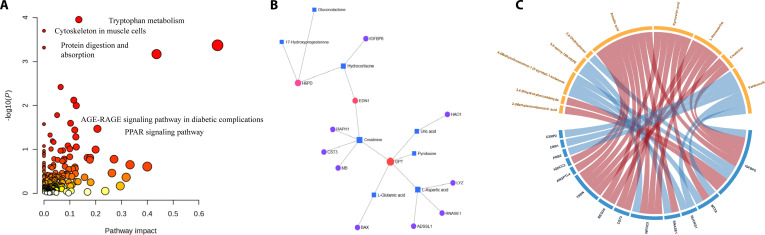
Integrative analysis of proteomic and metabolomic data in PAD with and without MACE. (A) Joint pathway enrichment analysis of differential proteins and metabolites. (B) Protein–metabolite interaction network. (C) Correlation analysis between differential proteins and metabolites. AGE-RAGE, advanced glycation end products and receptor for advanced glycation end products; PPAR, peroxisome proliferator-activated receptor; pathway impact, measure of the importance of the pathway in topology analysis.

To gain comprehensive mechanistic insights, we performed joint pathway enrichment analysis of differential metabolites and proteins using MetaboAnalyst 6.0. This integrative approach revealed significant perturbation of tryptophan metabolism in PAD patients who developed MACE, alongside notable enrichment of cytoskeleton organization, protein digestion and absorption, AGE-RAGE (advanced glycation end products and receptor for advanced glycation end products) signaling in diabetic complications, and PPAR signaling pathways (Fig. 4A). These findings suggest that amino acid dysregulation, muscle cytoskeletal remodeling, and chronic inflammation collaboratively contribute to the development of adverse events. Building on these pathway-level discoveries, we constructed a protein–metabolite interaction network (Fig. 4B) to further elucidate the molecular cross-talk underlying these systemic perturbations. Key metabolic intermediates—such as creatinine, pyridoxine, and L-glutamic acid—exhibited strong associations with proteins including GPT, CST3, and H6PD, emphasizing the interplay between renal function, amino acid utilization, and redox homeostasis in MACE risk. These network hubs primarily comprise metabolic enzymes and stress-response mediators rather than classical structural proteins, indicating that coordinated metabolic adaptation and cellular signaling may substantially impact clinical outcomes. Correlation and chord diagram analyses (Fig. 4C and Data S10) revealed statistical associations between differential proteins and metabolites. These findings should be interpreted as exploratory and hypothesis-generating; observed correlations do not imply direct functional or causal interactions, but may reflect systemic processes driven by shared upstream inflammatory or metabolic factors. Several matrix-modifying and energy-related proteins (CST3, BAX, and H6PD) and corresponding metabolic intermediates demonstrated highly concordant shifts, underscoring their potential utility as integrative biomarkers and therapeutic targets. Collectively, these integrative multiomics analyses provide a comprehensive perspective on the metabolic and proteomic disturbances underlying PAD progression. Rather than direct perturbation of structural proteins, our findings highlight the interconnected roles of metabolic dysregulation, cytoskeleton-related pathway activation, and chronic inflammatory and stress signaling in driving the increased risk of cardiovascular events.

### Recognition of prognostic-related proteins

**Fig. 5.**
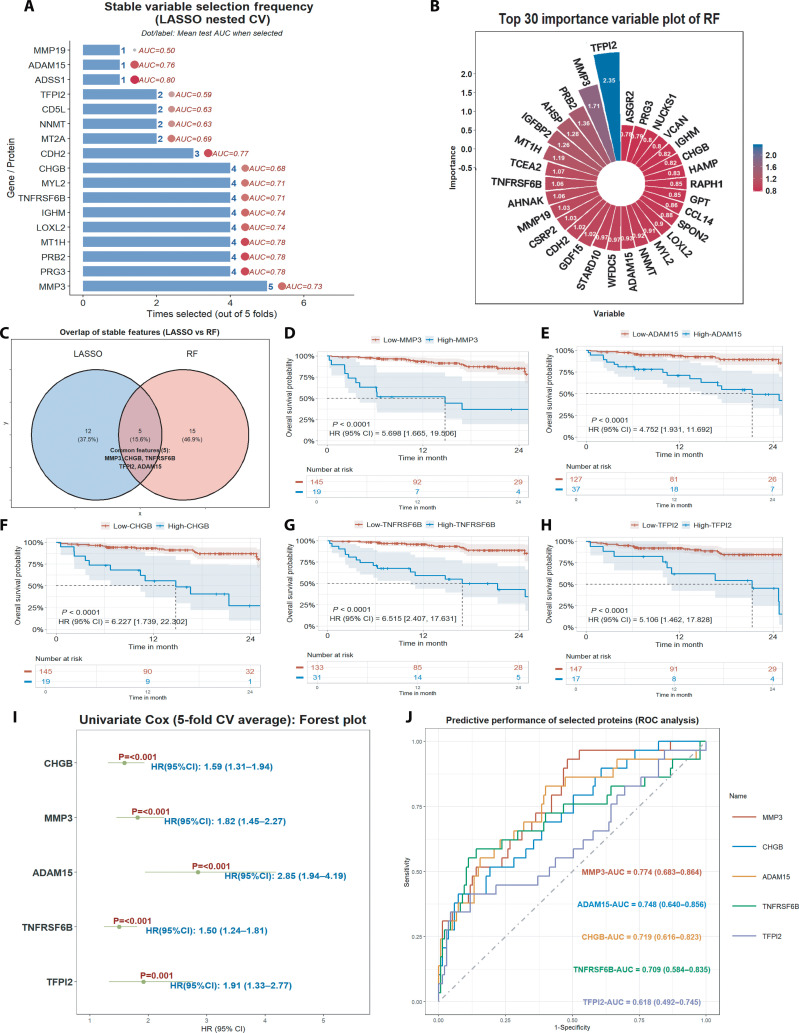
Identification and prognostic analysis of key proteomic predicting MACE occurrence. (A) Variable selection frequency of the top proteins from nested cross-validated LASSO regression, with mean test AUC indicated for each protein. (B) Top 30 variable importance plot from RF analysis on the training data, highlighting the most influential proteins. Color intensity represents variable importance. (C) Venn diagram showing the overlap of stable features identified by LASSO and RF approaches. (D to H) Kaplan–Meier survival curves for MACE-free survival, stratified by high vs. low expression of the 5 selected proteins (MMP3, ADAM15, CHGB, TNFRSF6B, and TFPI2). Log-rank *P* values and hazard ratios (HR [95% CI]) are reported. (I) Forest plot summarizing univariate Cox proportional hazards regression results for the 5 candidate proteins, with HRs and 95% confidence intervals (averaged across 5-fold cross-validation). (J) ROC curves illustrating the predictive performance of each selected protein for MACE, with area under the curve (AUC [95% CI]) for MMP3, ADAM15, CHGB, TNFRSF6B, and TFPI2. RF, random forest; LASSO, least absolute shrinkage and selection operator; AUC, area under the ROC curve; HR, hazard ratio; CI, confidence interval.

To systematically identify robust protein biomarkers predictive of MACE in PAD patients, we undertook rigorous statistical and machine learning analyses. Feature selection was performed using nested cross-validated least absolute shrinkage and selection operator (LASSO) regression, in which stability was summarized by selection frequency across 5-fold resamples (Fig. 5A), and random forest (RF) analysis of the training data, which highlighted top-ranked proteins based on overall variable importance (Fig. 5B). In addition, to further validate the robustness of our RF-based feature selection, a supplementary 5-fold cross-validated RF analysis was conducted and is presented in Fig S8. Integration of these models highlighted 5 proteins (CHGB, MMP3, ADAM15, TNFRSF6B, and TFPI2), as overlapping, stable candidates (Fig. 5C), which were subsequently prioritized for prognostic evaluation.

Kaplan–Meier survival analysis demonstrated that higher circulating levels of these proteins were each associated with significantly reduced MACE-free survival, with high-expression groups experiencing notably elevated event rates during follow-up (log-rank *P* < 0.0001, Fig. 5D to H). Univariate Cox regression confirmed that all 5 markers conferred increased risk for MACE, with effect sizes strongest for ADAM15 and all showing robust significance under cross-validation (Fig. 5I). We also assessed the discriminatory capacity of these proteins for predicting MACE using receiver operating characteristic (ROC) curve analysis (Fig. 5J). MMP3 demonstrated the highest predictive performance, achieving an area under the curve (AUC) of 0.774 (95% confidence interval [CI]: 0.683 to 0.864), followed closely by ADAM15 (AUC = 0.748, 95% CI: 0.640 to 0.856) and CHGB (AUC = 0.719, 95% CI: 0.617 to 0.823). TNFRSF6B and TFPI2 also provided moderate discrimination, with AUCs of 0.709 (95% CI: 0.584 to 0.835) and 0.619 (95% CI: 0.402 to 0.745), respectively. Collectively, these findings highlight a reproducible multiprotein signature with substantial potential for MACE risk stratification in PAD, underscoring the translational promise of integrating cross-validated machine learning and survival analysis approaches.

### Clinical indicator-based prognostic model

**Fig. 6.**
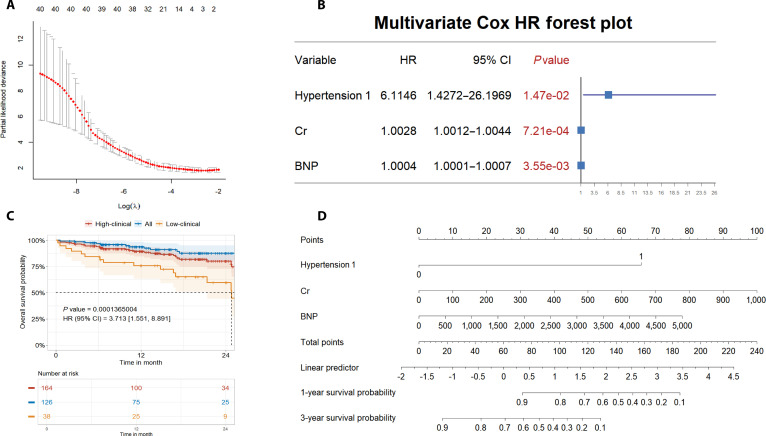
Construction of a clinical prognostic model for MACE in PAD patients. (A) Partial likelihood deviance plot for LASSO-Cox regression, illustrating the selection of optimal regularization parameter (λ) and identification of informative clinical predictors. (B) Multivariate Cox proportional hazards regression forest plot showing hazard ratios (HR) and 95% confidence intervals (CI) for each selected variable. (C) Kaplan–Meier survival curves for high- versus low-risk groups classified by the clinical risk score, demonstrating a significantly higher incidence of MACE in the high-risk group. (D) Clinical nomogram integrating hypertension, Cr, and BNP for individualized prediction of 1-year and 3-year survival probability in PAD patients. MACE, major adverse cardiovascular events; PAD, peripheral artery disease; LASSO, least absolute shrinkage and selection operator; HR, hazard ratio; CI, confidence interval; Cr, creatinine; BNP, B-type natriuretic peptide.

To establish a robust benchmark for multiomic predictive modeling, we first developed a clinical risk prediction model based exclusively on non-omics variables. LASSO regression was applied for dimensionality reduction and variable selection (Fig. 6A), with model stability assessed via 5-fold nested cross-validation. Hypertension, creatinine (Cr), and B-type natriuretic peptide (BNP) were robustly selected as the most informative clinical predictors of MACE, as further confirmed by their selection frequencies across cross-validation folds (Fig. S9). We subsequently performed multivariate Cox proportional hazards regression to assess the independent prognostic value of these variables. Hypertension (hazard ratio [HR] = 6.11; 95% CI: 1.43 to 26.97; *P* = 0.0147), Cr (HR = 1.0028; 95% CI: 1.0012 to 1.0044; *P* = 0.000721), and BNP (HR = 1.0004; 95% CI: 1.0001 to 1.0007; *P* = 0.00355) were identified as significant, independent predictors of MACE (Fig. 6B). To stratify risk, we calculated a composite clinical risk score. The optimal cutoff value for distinguishing high- and low-risk groups was determined by maximally selected rank statistics (Fig. S10). Kaplan–Meier survival analysis based on this cutoff demonstrated marked separation between high- and low-risk patients (Fig. 6C; log-rank *P* = 0.00014; HR = 3.713, 95% CI: 1.651 to 8.891), with the high-risk group showing a substantially increased incidence of MACE during follow-up. To facilitate individualized prognostic assessment, we constructed a clinical nomogram integrating hypertension, Cr, and BNP (Fig. 6D) for prediction of 1-year and 3-year survival probabilities. This clinical model offers a practical and interpretable framework for risk stratification and personalized management of PAD patients, and provides a reference benchmark for subsequent integrated multiomic modeling.

### Predictive modeling

**Fig. 7.**
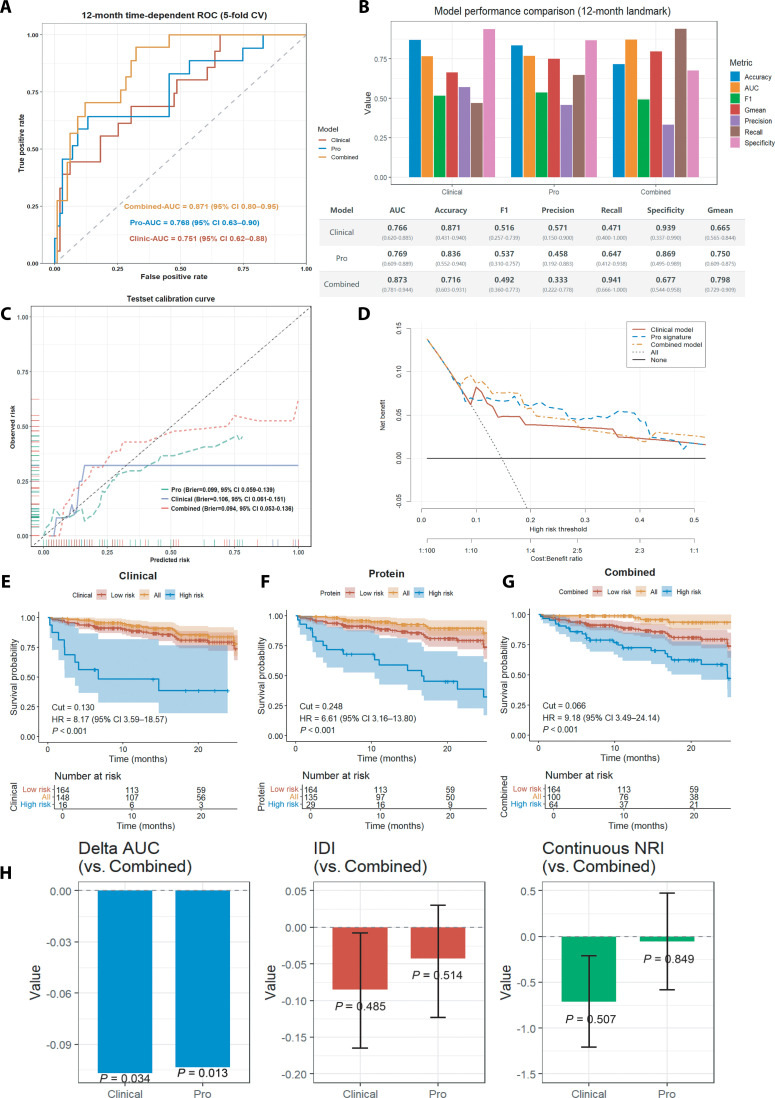
Comparative performance evaluation of the protein-based, clinical, and combined prognostic models for MACE prediction in PAD patients. (A) Time-dependent ROC curves at the 12-month landmark for the clinical (red), protein-based (pro, blue), and combined (orange) models, with AUC and 95% confidence intervals indicated. (B) Bar plot summarizing performance metrics including AUC, accuracy, F1 score, Gmean, precision, recall, and specificity for each model at 12 months. (C) Calibration curves for the clinical, protein, and combined models; Brier scores and 95% CI are shown, demonstrating good concordance between predicted and observed risk for the combined model. (D) Decision curve analysis showing the net clinical benefit of each model across varying risk thresholds for MACE. (E to G) Kaplan–Meier survival curves stratified by predicted risk groups (high vs. low) for the clinical model (E), protein model (F), and combined model (G), with cutoff values, hazard ratios (HR), 95% CI, and log-rank *P* values indicated. (H) Incremental model performance comparison by ΔAUC, IDI, and continuous NRI versus the combined model, with *P* values shown for each comparison. AUC, area under the curve; CI, confidence interval; HR, hazard ratio; PAD, peripheral artery disease; MACE, major adverse cardiovascular events; Pro, proteomic model; Clinical, clinical model; Combined, combined model; F1, F1 score; GMEAN, geometric mean; IDI, integrated discrimination improvement; NRI, net reclassification improvement.

To further improve risk stratification in PAD, we developed a protein-based prognostic signature and systematically compared 3 models: a clinical model, a protein-based model, and an integrated model combining clinical and proteomic features. Model discrimination was evaluated using 5-fold cross-validation with out-of-fold prediction. At a fixed 12-month landmark, time-dependent ROC analysis (Fig. 7A) demonstrated that the integrated model achieved the highest discriminative performance, with a 12-month AUC of 0.873 (95% CI: 0.781 to 0.944), outperforming the protein-only (AUC = 0.769, 95% CI: 0.609 to 0.889) and clinical models (AUC = 0.766, 95% CI: 0.620 to 0.885). Fold-wise AUC distributions and corresponding ROC curves are presented in Figs. S11 to S16, illustrating the performance stability across resamples. Comprehensive model performance metrics at the 12-month landmark with Accuracy = 0.716 (95% CI: 0.603 to 0.931), F1 score = 0.492 (95% CI: 0.360 to 0.773), Precision = 0.333 (95% CI: 0.222 to 0.778), Recall = 0.941 (95% CI: 0.666 to 1.000), Specificity = 0.677 (95% CI: 0.544 to 0.958), and Gmean = 0.798 (95% CI: 0.729 to 0.909) are summarized in Fig. 7B, further highlighting the superior performance of the integrated model. Assessment of calibration (Fig. 7C) revealed good concordance between predicted and observed risk at 12 months for the integrated model, and decision curve analysis (Fig. 7D) showed a higher net clinical benefit across a range of threshold probabilities. Kaplan–Meier survival analyses (Fig. 7E to G) were used to visualize risk stratification based on the optimal cutoff derived from the 12-month landmark ROC analysis. These curves demonstrate clear separation between high- and low-risk groups during the entire follow-up period. These analyses demonstrate clinical separation between risk groups. Incremental model performance was further assessed using ΔAUC, integrated discrimination improvement (IDI), and continuous net reclassification improvement (NRI) (Fig. 7H). The integrated model showed a significant improvement in AUC compared with the clinical (*P* = 0.034) and protein-only models (*P* = 0.013), while IDI and continuous NRI showed numerical but nonsignificant improvements.

## Discussion

In this study, we employed a multiomics strategy combining proteomics and metabolomics to systematically analyze the molecular mechanisms and risk stratification of MACE in patients with PAD. Through the integrated analysis of proteomic and metabolomic data, we deeply explored the synergistic effect between protein dysregulation and metabolic changes, and these interrelated molecular alterations collectively exacerbated the cardiovascular risk in PAD patients. The study revealed core mechanism pathways such as ECM remodeling, inflammatory response, and metabolic reprogramming. Furthermore, we developed an efficient prognostic model based on plasma proteomic profiles, providing a solid scientific foundation for the precise prediction and individualized intervention of cardiovascular outcomes related to PAD. These innovative findings not only enriched the academic understanding of the disease,s molecular mechanisms but also promoted the clinical translation and application of high-risk population stratification and targeted therapy.

A principal mechanistic finding was the consistent up-regulation of matrix metalloproteinases MMP3 and MMP19 in PAD patients who subsequently experienced MACE. MMP3 has a well-documented role in ECM hydrolysis, particularly degrading vascular collagen and elastin, thereby fueling plaque destabilization and vessel wall injury [11,12]. Although MMP19 remains less extensively investigated, emerging evidence now links it to pathological vascular remodeling and chronic inflammation—characteristics integral to advanced atherosclerosis [13–15]. It is noteworthy that both MMP3 and MMP19 were deeply embedded within broader regulatory networks, interacting extensively with other ECM and signaling proteins. This network integration further underscores their functional significance in high-risk patients. The up-regulated expression of these metalloproteinases may serve as early molecular biomarkers for the identification of high-risk individuals and represent promising therapeutic targets for the prevention of adverse cardiovascular events. This highlights the potential clinical value of targeting key nodes within the proteolytic and inflammatory cascades in PAD management.

In addition, proteins such as NID2 and CHGB were found to be associated with MACE risk. NID2 is essential for ECM structure and vascular integrity, and its dysregulation has been linked to vascular disease progression [16,17]. CHGB is implicated in vascular tone regulation and inflammatory pathways, both key in cardiovascular event pathogenesis [18,19]. Collectively, these proteins reflect a complex network of inflammation, vascular damage, and ECM remodeling underlying increased cardiovascular risk in PAD [20,21].

At the metabolic level, our data revealed pronounced dysregulation of amino acid, organic acid, and fatty acid metabolic pathways, with up-regulated indolepyruvate, γ-aminobutyric acid (GABA), and creatinine as prominent features. These changes were associated with neuroinflammation, immune modulation, and metabolic dysfunction—all hallmarks of cardiovascular diseases in PAD [22]. Indolepyruvate, a tryptophan metabolite, serves as a bridge between immune activation and oxidative stress—hallmarks of plaque instability [23,24]. GABA, while classically a neurotransmitter, has increasingly been recognized for its immune-modulatory and vascular inflammatory effects [25]. These metabolic shifts, visualized in integrated molecular networks, reveal direct cross-talk with ECM proteins, particularly those involved in tissue remodeling and inflammatory signaling.

It is important to note that among the top metabolic features identified in our study are creatinine and certain amino acid metabolites. These signals may reflect the severity of underlying renal or cardiac dysfunction in addition to their association with MACE risk. While sensitivity analyses adjusting for major comorbidities largely confirmed the primary findings, some key metabolic features may still capture underlying organ dysfunction rather than being specific to MACE. We did not adjust the integration analyses for baseline renal or cardiac biomarkers (e.g., creatinine or BNP), as these variables may act as mediators or represent components of the disease process itself; therefore, residual confounding cannot be fully excluded. These observations underscore the intertwined nature of organ dysfunction and cardiovascular risk, and highlight the need for cautious interpretation of omics findings in complex clinical populations.

Our integrative multiomics analysis provides crucial molecular insights into the mechanisms that drive MACE in patients with PAD. Results revealed highly dynamic molecular interactions among inflammation, ECM remodeling, and metabolic reprogramming. Key proteins such as IGFBP6, CST3, and RNASE1 were closely associated with central metabolites including creatinine, pyridoxine, uric acid, L-glutamate, and L-aspartate, with these molecules collectively implicated in amino acid metabolism, energy homeostasis, and redox signaling [24,26–30]. Pathway enrichment and network analyses further highlighted significant disturbances in tryptophan metabolism, AGE-RAGE signaling, and cytoskeletal regulation, with protein–metabolite interactions extending to neuroinflammation and immune modulation, reflecting the complex, multifactorial nature of atherosclerotic progression in high-risk PAD patients [31,32].

Based on these findings, we discovered the “inflammation-structure-metabolism” feedforward disease pattern. Persistent inflammation induces expression changes in proteins such as IGFBP6, CST3, and EDN1, thereby facilitating ECM remodeling and vascular structural instability [33]. This structural deterioration further enhances immune cell infiltration and metabolic stress, and metabolic disturbances then feedback to amplify cellular inflammation and matrix remodeling, establishing a self-reinforcing cycle that increases the risk of plaque instability and MACE [34]. Our multiomics integration not only illustrates the synergistic involvement of protein–metabolite axes in pathogenic processes but also demonstrates tight cross-regulation among amino acid turnover, fatty acid metabolism, glycolysis, and related pathways. This systems biology approach deepens our understanding of PAD mechanisms and underscores the potential of integrative omics for precision biomarker identification and therapeutic target discovery in high-risk vascular disease [35].

Moreover, with the continual advancement of affinity-based high-throughput platforms and mass spectrometry technologies, the quantitative measurement of thousands of plasma proteins has become feasible, greatly facilitating the discovery of novel biomarker panels for clinical risk prediction [36]. In recent years, proteomic biomarkers have garnered increasing attention in cardiovascular research and risk stratification. Large-scale cohort studies have demonstrated that proteomics-based risk models can markedly enhance the prediction of cardiovascular events and mortality, outperforming traditional clinical risk factors [37–39]. Building upon these mechanistic insights and advances in the field, we further developed a prognostic model based on proteomic features.

In our study, this proteomics-based model demonstrated high discriminatory accuracy for predicting MACE (AUC > 0.80), consistent with recent multicenter studies employing the plasma proteome for clinical risk stratification. Notably, when applied to a PAD cohort, our multivariable proteomic model identified MMP3, MMP19, NID2, and CHGB as key components for MACE risk prediction. Compared to single-biomarker measurements or conventional laboratory indices, multiplex proteomic profiling offers superior sensitivity and specificity, as it can simultaneously capture a range of pathophysiological processes—including inflammation, ECM remodeling, vascular integrity, and metabolic adaptation—within a unified molecular fingerprint. Collectively, our findings underscore the transformative potential of proteomics for personalized prognostication and early intervention in cardiovascular disease.

Given these promising results, biomarker-based risk assessment has the potential to enhance PAD management by enabling more precise identification of individuals at high risk for MACE than traditional clinical factors alone. In clinical settings, such stratification could support clinicians in tailoring treatment intensity and follow-up schedules, ensuring that high-risk patients receive closer surveillance or timely therapeutic adjustments, while low-risk patients may avoid unnecessary interventions. These approaches may facilitate more efficient allocation of healthcare resources and promote individualized care. Nevertheless, widespread adoption will require further validation in large, diverse patient cohorts and integration of biomarker testing into routine clinical workflows. Future studies should also assess whether these strategies can improve clinical outcomes and cost-effectiveness in real-world practice.

### Limitations

This study has several notable limitations that should be taken into account. First, the sample size and number of outcome events were limited, and all participants were enrolled from a single center. These factors may restrict the statistical strength of our findings and increase the risk of overfitting, particularly given the complexity of the multiomics analyses. Second, our study specifically targeted hospitalized PAD patients who underwent endovascular revascularization. Because of this inclusion criteria, our cohort contained a higher proportion of individuals with advanced or severe disease. Therefore, our study population does not cover the full clinical spectrum of PAD, including those with milder cases typically seen in outpatient or community settings. As a result, the generalizability of our risk model to the broader PAD population, especially patients with mild to moderate disease, remains uncertain. The robustness and universal applicability of these findings will require further validation in larger, more diverse, and multicenter cohorts that include the full range of PAD severity. Third, our predictive models were developed and tested within the same study cohort, without validation using independent external data. This limits confidence in the generalizability and robustness of the identified proteomic signatures, and further multicenter validation studies are required. Fourth, while our integrated omics approach revealed several potentially relevant biological pathways, the study is primarily observational and relies on association rather than direct mechanistic evidence. Future experimental studies, including cell or animal models and tissue-specific analyses, will be necessary to determine causal relationships and clarify the origins of circulating biomarkers. Fifth, from a practical standpoint, mass spectrometry-based biomarker panels are not yet feasible for routine clinical use due to cost and technical requirements. The incremental predictive gain observed in this study must be balanced against these logistical barriers before clinical adoption can be considered. Finally, our study is limited by its modest sample size and relatively short follow-up duration. Although most adverse events occurred after the perioperative window, a small number did occur early post-intervention and may reflect perioperative as well as long-term cardiovascular risk. Due to limited case numbers, we could not reliably separate early and late events for further analysis. These factors may influence the distinction between perioperative and chronic cardiovascular outcomes. Nonetheless, our findings lay the groundwork for future studies with larger sample sizes and extended follow-up to better address these distinctions.

## Conclusion

In summary, our prospective multiomics study in patients with PAD identified significant proteomic and metabolomic alterations associated with MACE. These changes implicate key pathways of ECM remodeling, immune activation, and metabolic dysregulation in adverse outcomes, revealing potential mechanistic links and novel biomarker candidates. By integrating clinical variables with proteomic features, we established a robust prognostic model that outperformed both traditional clinical models and those based solely on proteomics in predicting long-term cardiovascular risk. These findings underscore the transformative potential of proteomics and metabolomics for personalized risk stratification and early intervention in PAD. Further studies with larger external cohorts and functional validation are warranted to translate these discoveries into clinical practice.

## Materials and Methods

### Study population

This study was approved by the Ethics Committee of Beijing Hospital, and written informed consent was obtained from all participants prior to enrollment. We conducted a single-center, prospective cohort study, consecutively enrolling 175 adult inpatients with symptomatic, angiographically confirmed PAD. All included patients were scheduled for unilateral or bilateral lower-extremity endovascular revascularization during their hospitalization. Every patient meeting the eligibility criteria underwent comprehensive baseline data collection and omics profiling. Fasting venous blood samples were collected within 12 h of hospital admission for omics analysis (see Fig. 1). Baseline medication use, including anticoagulant and antiplatelet therapy, was systematically documented prior to blood sampling. Any in-hospital peri-procedural antithrombotic or high-dose therapy was initiated only after blood sampling.

Of the 175 patients initially enrolled, 11 were excluded from the final analysis due to missing follow-up data, resulting in 164 patients included in longitudinal outcome analysis (135 non-MACE, 29 MACE; see Fig. S1 for the detailed flow diagram). The diagnosis of PAD was made in accordance with the latest guidelines from the American College of Cardiology/American Heart Association (2024), and was further confirmed by angiographic evidence of ≥70% cross-sectional area stenosis in the iliac arteries or below [40]. To minimize confounding, we strictly excluded patients with conditions that could affect vascular calcification or the expression of inflammatory mediators, including active infection, cardiovascular events within the preceding month, major surgery or severe trauma (such as untreated fractures) within the past 3 months, or autoimmune diseases.

### Clinical parameter characterization

We collected each patient’s baseline demographic information, including sex, age, and medication history and comorbidities such as hypertension, diabetes, malignancy, and coronary artery disease. Clinical laboratory parameters were systematically obtained at admission. All clinical variables were independently processed and manually standardized by 2 experienced physicians, with interrater consistency checks performed to ensure data reliability and minimize information bias.

### Plasma collection, processing, and storage

All participants underwent fasting venous blood sampling within 12 h of hospital admission. Peripheral blood (2 ml) was collected into EDTA anticoagulant tubes, typically in the morning or forenoon, and was gently inverted several times for thorough mixing. Plasma was separated by centrifugation at 3,000 rpm for 10 min at 4 °C, within 1 h if kept at room temperature or within 2 h if kept at 4 °C. The supernatant plasma was carefully transferred into cryovials and stored at −80 °C until proteomic and metabolomic analyses. Any plasma samples with visible hemolysis were excluded from downstream analyses to ensure sample quality.

### Follow-up and endpoint events

Patients were advised to undergo postoperative follow-up assessments at 1, 3, and 6 months, and annually thereafter, using vascular imaging modalities such as ultrasound, computed tomography angiography (CTA), or digital subtraction angiography (DSA). Follow-up information was collected through structured review of medical records and direct telephone interviews when necessary. The follow-up endpoint was defined as the earliest of the following: occurrence of MACE, date of last contact, or the last follow-up date (June 2025). Adjudication of endpoint events was performed by a trained vascular surgeon using prespecified criteria, based on comprehensive review of clinical records and structured follow-up interviews. The primary outcome analyzed was MACE, defined as a composite of nonfatal myocardial infarction, nonfatal stroke, or cardiovascular death occurring at any time from the first postoperative day until the end of follow-up. All MACE events identified during this time frame were included in the analysis. To ensure consistency and adequate event observation, all included patients had a minimum follow-up duration of 6 months.

### Proteomic analysis

Plasma samples (100 μl, post-centrifugation) were incubated with prewashed PTM-Max magnetic nanoparticles (PTM Bio, Hangzhou, China) at 1,200 rpm and 37 °C for 1 h. After 3 washes, on-bead tryptic digestion was conducted in 150 μl of digestion buffer containing trypsin (10 ng/μl) at 37 °C overnight. Protein disulfide bonds were reduced with 5 mM DTT at 56 °C for 30 min and alkylated with 11 mM iodoacetamide (IAM) at room temperature for 15 min in the dark. The resulting peptides were purified using C18 ZipTips, vacuum-dried, and stored at −80 °C until analysis. Prior to liquid chromatography–tandem mass spectrometry (LC–MS/MS)analysis, samples were reconstituted in 0.1% formic acid. Peptides were separated on a Vanquish Neo UHPLC system (Thermo Fisher Scientific) using a short reversed-phase gradient (total runtime 7.6 min) and analyzed on an Orbitrap Astral mass spectrometer. Data were acquired in data-independent acquisition (DIA) mode, and no additional HPLC-based peptide fractionation was performed. All plasma samples were processed and analyzed in a single analytical batch using identical LC–MS settings. We additionally performed standard quality control (QC) assessments (Pearson correlation, PCA, and total intensity distribution checks), which did not indicate processing-order trends or technical drift. Detailed LC–MS/MS acquisition parameters are provided in the Supplementary Methods.

DIA data were processed using Spectronaut (v18, Biognosys). Tandem mass spectra were searched against the UniProt *Homo sapiens* reference proteome (release 2024 December 2) combined with a reverse decoy database. Trypsin/P was specified as the digestion enzyme, allowing up to 2 missed cleavages. Carbamidomethylation of cysteine was set as a fixed modification, while protein N-terminal acetylation and methionine oxidation were set as variable modifications. After data filtering, missing values were imputed using the K-nearest neighbors (KNN) algorithm, and protein-level abundances were center-normalized.

### Metabolomic analysis

Plasma samples were extracted with a methanol–acetonitrile mixture containing internal standards, followed by low-temperature sonication, protein precipitation, centrifugation, and nitrogen drying. After reconstitution, supernatants were analyzed by LC-MS using a UHPLC-Q Exactive HF-X system (Thermo Fisher Scientific) equipped with an ACQUITY UPLC HSS T3 column, in both positive and negative ion modes. QC samples were prepared by pooling equal aliquots from all samples and were injected regularly throughout the batch to monitor data quality and instrument stability. Further details are provided in the Supplementary Methods.

Raw data were processed using Progenesis QI software (Waters) for peak detection, alignment, and peak area extraction. Metabolites were identified by matching accurate mass and MS/MS spectra to public databases, and metabolite identifications were treated as putative annotations (MSI level 1 was not claimed). Features detected in at least 80% of samples were retained. Missing values were imputed using the minimum value, and peak intensities were normalized by sum normalization. This sum normalization step was applied to reduce errors introduced by sample preparation and instrument instability, yielding the normalized data matrix. To ensure analytical stability, features with QC relative standard deviation > 30% were removed.

### Statistical analysis

Data were analyzed using R software. For continuous variables, data are presented as mean ± standard deviation or median (interquartile range), and comparisons between groups were performed using Student *t* test or Mann–Whitney *U* test, as appropriate. Categorical variables were compared using chi-square or Fisher’s exact test. All statistical tests were 2-sided, with a significance level set at *P* < 0.05. The performance of the models was assessed using AUC, HR, Cox regression, Brier score, and Kaplan–Meier analysis.

### Proteomics data processing and statistical analysis

Differential protein expression analysis was performed using linear models with the limma package and empirical Bayes moderation. Proteins were identified as significantly differentially expressed based on a false discovery rate (FDR) adjusted *P* value < 0.05 and an absolute log2 fold change threshold of 0.58. FDR was controlled by the Benjamini–Hochberg (BH) procedure unless otherwise specified. Results were visualized using volcano and bar plots to illustrate overall expression changes, and radar plots were used to summarize abundance and fold change for the top 20 significant proteins. Significantly altered proteins were subjected to Gene Ontology and KEGG pathway enrichment analyses using the clusterProfiler package. Gene identifiers were mapped to Entrez or UniProt IDs as needed for functional annotation. The most relevant pathways were visualized using bar plots and chord diagrams. To further illustrate the overlap between key biological processes and proteins, category–protein association networks were generated, highlighting relationships among the top enriched terms and core proteins.

To assess the robustness of the proteomic findings and mitigate potential confounding, sensitivity analyses were conducted in which preadmission antithrombotic medication use and major baseline comorbidities (chronic kidney disease, malignancy, and hypertension) were included as covariates in the linear models.

### Metabolomics data processing and statistical analysis

Differential metabolite abundance analysis was performed using linear models with the limma package and empirical Bayes moderation to compare metabolite levels between PAD patients with and without MACE. *P* values were adjusted using the BH procedure, and metabolites were considered significantly differentially abundant at FDR < 0.05 and |log2 fold change| > 1. PLS-DA was performed as a supervised exploratory method using the mixOmics package, and VIP scores were calculated to rank metabolites contributing to group separation. Model performance was evaluated using cross-validated *R*^2^/*Q*^2^, and permutation testing was used to assess potential overfitting. The chemical class composition of differential metabolites was summarized using pie charts. Functional and pathway enrichment analyses were performed using MetaboAnalyst 6.0, with FDR < 0.05 set as the threshold for pathway significance. KEGG pathway mapping, enrichment ratio calculation, interaction bubble plots, and network graphs were derived using integrated MetaboAnalyst modules, based on metabolite annotation and differential expression results. All statistical analyses and data visualizations were performed in R, with graphical outputs rendered using ggplot2 and mixOmics, and pathway/network diagrams generated in MetaboAnalyst 6.0.

Similarly, sensitivity analyses were performed by including preadmission antithrombotic medication status and major clinical comorbidities as covariates in the differential abundance models to evaluate and control for potential confounding effects. Metabolite identifications were treated as putative annotations (MSI level 1 was not claimed) based on accurate mass and MS/MS spectral similarity to public databases.

### Multiomics integration

To elucidate the molecular mechanisms underlying increased cardiovascular risk in PAD patients, we performed joint analysis of differentially expressed proteins and metabolites. Pathway enrichment was performed using the Joint Pathway Analysis module of MetaboAnalyst 6.0. Protein–metabolite association networks were constructed in MetaboAnalyst to visualize cross-omic connections. Pairwise Pearson correlations between selected proteins and metabolites were calculated in R (v4.3), with correlations (|*r*| > 0.65, FDR < 0.05) visualized using chord diagrams generated by the circlize package. Multiple-testing correction was applied in all analyses using the BH procedure, with statistical significance defined as FDR < 0.05.

### Feature selection via machine learning

To identify circulating protein biomarkers predictive of MACE in PAD patients, multiple machine learning approaches were applied using R (version 4.3.0). A 5-fold cross-validation framework was implemented to evaluate feature selection stability and model performance. In each fold, samples were partitioned into training and testing subsets, with feature selection procedures conducted exclusively on the training data. LASSO regression modeling was conducted using the glmnet package. Within each training fold, an inner cross-validation procedure (cv.glmnet) was used to select the optimal regularization parameter (lambda). Features with nonzero coefficients at the optimal lambda were retained for that fold. Feature stability was assessed by summarizing the selection frequency of each protein across cross-validation folds, and proteins consistently selected across folds were considered stable candidates.

RF models were built using the randomForest package, and variable importance was calculated based on the mean decrease in Gini index and classification accuracy. RF feature importance was evaluated within the same 5-fold cross-validation framework, and feature stability was summarized across folds based on Gini importance rankings. Venn diagram visualizations of overlapping top features were produced using the ggVennDiagram package. For details on feature selection algorithms and literature references, see Table S7.

To address the issue of dimensionality and minimize the risk of model overfitting given the limited number of outcome events, we applied a dimensionality reduction strategy for the proteomic data prior to multivariable regression. Specifically, the machine learning-based feature selection above was used to select core protein candidates, which were subsequently combined into a composite protein risk score. This aggregated score was treated as a single variable in the final multivariable Cox regression model, alongside selected clinical covariates (hypertension, creatinine, and BNP). As a result, the final risk prediction model included a total of 4 predictors, yielding an events-per-variable (EPV) ratio of approximately 7.25 (29 events/4 variables), which satisfies the commonly accepted minimum threshold of 5 EPV for stable estimation in small cohorts [41].

### Clinical variable selection and risk modeling

Clinical non-omics predictors were selected by LASSO regression using the glmnet package with 5-fold cross-validation. Multivariable Cox proportional hazards regression models were fitted using the survival package, generating HRs, 95% CIs, and *P* values for each variable. Model discrimination was evaluated at a fixed 12-month landmark using time-dependent ROC analysis to account for right censoring.

### Survival analysis and validation

Kaplan–Meier survival curves were generated for risk groups defined by model-derived 12-month predicted risk scores using the survminer package, and significance was evaluated by log-rank tests. Cox proportional hazards models were used to estimate HRs and 95% CIs between predefined risk groups, serving as supportive evidence for model-based risk stratification. Forest plots for HRs and CIs were visualized by forestplot. Cutoff values for risk scores were derived in a data-driven manner within the internal validation framework using predicted 12-month risk scores. Nomograms were constructed for visualization of model structure using the rms package.

### Integrated multiomic prognostic model comparison

Predictive models were constructed using clinical variables, proteomic features, and an integrated model combining both sets of predictors. Model discrimination was evaluated using time-dependent ROC curves at the 12-month landmark with 5-fold cross-validation, and performance was quantified by the AUC. Within the cross-validation framework, fold-specific AUCs were calculated on held-out samples and summarized as mean AUC to assess the stability of model discrimination across resamples.

Additional classification performance metrics, including accuracy, sensitivity, specificity, precision, recall, F1 score, and geometric mean (Gmean), were calculated at the same time point. Ninety-five percent CIs for AUC and classification metrics were estimated using bootstrap resampling (1,000 iterations). Bootstrap resampling was applied to cross-validated predictions to characterize uncertainty in performance estimates.

Model calibration was assessed using calibration curves and the Brier score at the 12-month horizon. Decision curve analysis was performed to evaluate the net clinical benefit of each model across a range of risk thresholds. For risk stratification, the optimal cutoff from the 12-month landmark ROC analysis was used to assign patients into high- and low-risk groups, followed by Kaplan–Meier curves for all patients throughout the follow-up period. HRs and 95% CIs were calculated via Cox regression. Incremental predictive performance of the integrated model relative to the clinical and protein-only models was further assessed using ΔAUC, IDI, and continuous NRI. All survival analyses and visualizations were conducted using the survminer package.

## Data Availability

Readers interested in the raw metabolomics and proteomics data may contact the corresponding authors. Access to the data is limited to noncommercial research purposes and must comply with relevant Chinese laws and regulations. All authors who use data or information from this study are required to cite this publication.

## Appendix A – VIP-PAD Study Collaborators

Yongjun Li^a^, Zuoguan Chen^a^, Wenxin Zhao^a^, Xiaolu Li^a,^^b^, Yifan Cao^a^, Bowen Zhang^a^, Yaming Guo^a,^^c^, Shang Gao^a^, Xihao Zhang^a^, Yongpeng Diao^a^, Yong Lan^a^, Peng Li^a^, Jiyang Wang^a^, Shuping Tan^a^, Chengran Lu^a^, Zhengxi Xu^a^, Zhiyuan Wu^a^, Yuqing Miao^a^, Cheng Ni^a^, Yixuan Wang^a^, Yixuan Yang^a^, Ning Zhao^a^, Yaping Feng^b^, Hai Feng^c^, Lishan Lian^c^, Sheng Yan^d^, Yifei Li^d^, Qiang Zhang^d^, Tao He^e^, Shaobo Cao^e^, Qiang Guan^f^, Hongyong Duan^f^, Yunfeng Zhang^f^, Tian Wei^f^, Hongpu Li^g^, Fan Zhang^g^, Bin Wang^h^, Hongcheng Ren^h^, Zhiyong Zhou^i^, Cheng Peng^i^, Hualei Bai^i^, Huaping Wu^j^, Li Zhang^j^, Wei Ying^j^, Tao Zhou^k^, Longxiang Zhang^k^, Guiming Wang^l^, Liuming Shi^l^, Chenglong Fang^m^

^a^Department of Vascular Surgery, Beijing Hospital, National Center of Gerontology; Institute of Geriatric Medicine, Chinese Academy of Medical Sciences, P.R. China.

^b^Department of Vascular Surgery, Beijing Tongren Hospital, Capital Medical University, Beijing, P.R. China.

^c^Department of Vascular Surgery, Beijing Friendship Hospital, Capital Medical University, Beijing, P.R. China.

^d^Department of Vascular Surgery, Second Hospital of Shanxi Medical University, Taiyuan, Shanxi, P.R. China.

^e^Department of Vascular Surgery, The Central Hospital of Wuhan, Tongji Medical College, Huazhong University of Science and Technology, Wuhan, P.R. China.

^f^The Vascular Surgery Department of Shanxi Provincial People’s Hospital, Taiyuan, P.R. China.

^g^The Fifth Clinical Medical College of Henan University of Chinese Medicine (Zhengzhou People’s Hospital), Zhengzhou, P.R. China.

^h^Department of Interventional Vascular, Aerospace Center Hospital, Beijing, P.R. China.

^i^Xing’an League People’s Hospital, P.R. China.

^j^Department of Vascular Surgery, Dazhou Central Hospital, Dazhou, Sichuan, P.R. China.

^k^Peripheral vascular department (wound repair), The First Affiliated Hospital of Henan University of Chinese Medicine, Zhengzhou, P.R. China.

^l^Department of Vascular Surgery, First Hospital of Shanxi Medical University, Taiyuan, P.R. China.

^m^Provincial Hospital, Fuzhou University Affiliated Provincial Hospital, Fuzhou, Fujian, P.R. China.
