# Supplementary material for: Prediction of Major Adverse Cardiovascular Events in Peripheral Artery Disease: Integrating Metabolomics and Proteomics for Risk Stratification
Source: Research (Wash D C). 2026 May 6;9:1229. doi: 10.34133/research.1229 (PMC13148185; doi:10.34133/research.1229)
Supplement: Supplementary 1 — Supplementary Methods Tables S1 to S7 Figs. S1 to S16 Data S1 to S10 [file research.1229.f1.zip › supplment table and figure R1.pdf]

Supplementary Materials

Supplementary Methods 3

S1 Proteomic Analysis 3

S2 Metabolomic Analysis 5

Supplementary Tables 7

Table S1. Top 30 differentially expressed proteins between PAD patients with and without MACE 7

Table S2. Top 30 differentially expressed metabolomic between PAD patients with and without MACE 8

Table S3. Sensitivity analysis results for the top 30 differentially expressed proteins after adjustment for pre-admission antithrombotic medication use 9

Table S4. Sensitivity analysis results for the top 30 differentially expressed metabolomic after adjustment for pre-admission antithrombotic medication use 10

Table S5. Sensitivity analysis results for the top 30 differentially expressed proteins after adjustment for major baseline comorbidities 11

Table S6. Sensitivity analysis results for the top 30 differentially expressed metabolomic after adjustment for major baseline comorbidities 12

Table S7. Overview of the main algorithms used for feature selection in this study and relevant literature references 13

Supplementary Figures 14

Figure S1. Patient flow diagram 14

Figure S2. Pearson correlation matrix across samples 15

Figure S3. Principal component analysis (PCA) of proteomic profiles for all samples 16

Figure S4. Distribution of proteomic signal intensities across patient groups 17

Figure S5. Permutation-based validation of PLS-DA model using balanced error rate (BER) 18

Figure S6. Classification error rates for PLS-DA components 19

Figure S7. Variance explained by PLS-DA components.. **20**
Figure S8. Stable feature selection by random forest (RF) using Gini importance.... **21**
Figure S9. Stability of clinical variable selection by LASSO-Cox regression……..... **22**
Figure S10. Determination of the optimal cutoff for clinical risk score stratification. **23**
Figure S11. Fold-wise 12-month AUCs for the clinical model................................. **24**
Figure S12. Fold-wise ROC curves for the clinical model at 12 months.................... **25**
Figure S13. Fold-wise 12-month AUCs for the protein-based model......................... **26**
Figure S14. Fold-wise ROC curves for the protein-based model at 12 months.......... **27**
Figure S15. Fold-wise 12-month AUCs for the combined clinical and proteomic model………………………………………………………………………….. **28**
Figure S16. Fold-wise ROC curves for the combined clinical and proteomic model at 12 months………………………………………………………………………….. **29**

Supplementary Methods

S1 Proteomic Analysis

1.1 Plasma Sample Preparation

Post-centrifuged plasma samples (100 µL) were incubated with pre-washed PTM-Max magnetic nanoparticles (PTM Bio, Hangzhou, China) at 1200 rpm and 37 °C for 1 h, followed by three washing steps. On-bead digestion was performed in 150 µL digestion buffer with trypsin (10 ng/µL) at 37 °C overnight. Proteins were reduced with 5 mM dithiothreitol at 56 °C for 30 min and alkylated with 11 mM iodoacetamide at room temperature for 15 min in the dark. Peptides were desalted using C18 ZipTips, vacuum-dried, and stored until LC–MS analysis. No isobaric labeling, HPLC-based peptide fractionation, or affinity enrichment was performed.

1.2 LC–MS/MS Analysis

1.2.1 Liquid Chromatography

Tryptic peptides were reconstituted in 0.1% formic acid and separated on a home-made reversed-phase analytical column (15 cm length × 100 µm inner diameter) using a Vanquish Neo UHPLC system (Thermo Fisher Scientific) at a constant flow rate of 300 nL/min. Mobile phases consisted of solvent A (0.1% formic acid in water) and solvent B (0.1% formic acid in 80% acetonitrile/water). The LC gradient was as follows: 0–1.6 min, 4–22.5% B; 1.6–2.0 min, 22.5–35% B; 2.0–2.6 min, 35–55% B; 2.6–2.7 min, 55–99% B; 2.7–7.6 min, 99% B.

1.2.2 Mass Spectrometry and DIA Acquisition

Separated peptides were analyzed on an Orbitrap Astral mass spectrometer equipped with a nano-spray ionization source. The spray voltage was set to 1900 V. Precursor ions were analyzed in the Orbitrap analyzer, and fragment ions were analyzed in the Astral analyzer. Full MS scans were acquired over an m/z range of 480–780 at a resolution of 240,000. MS/MS scans were acquired with a fixed first mass of 150 m/z at a resolution of 80,000. Higher-energy collisional dissociation (HCD) was performed at a normalized collision energy (NCE) of 25%. Automatic gain control

(AGC) was set to 500%, with a maximum injection time of 3 ms.

Data were acquired in data-independent acquisition (DIA) mode. Following each full MS scan, precursor ions were sequentially fragmented using multiple consecutive m/z isolation windows covering the defined precursor mass range. The detailed DIA window scheme was defined in the instrument acquisition method.

1.3 Database Search and Protein Quantification

DIA data were processed using Spectronaut software (version 18, Biognosys) with the embedded Pulsar search engine and default parameters. Tandem mass spectra were searched against the Homo_sapiens_9606_SP_20241202.fasta database (20,422 entries), concatenated with a reverse decoy database. Trypsin/P was specified as the digestion enzyme, allowing up to two missed cleavages. Carbamidomethylation of cysteine was set as a fixed modification, while oxidation of methionine and protein N-terminal acetylation were set as variable modifications. False discovery rates (FDRs) were controlled at 1% at the protein, peptide, and peptide-spectrum match (PSM) levels. Protein identification required at least one unique peptide.

Data quality and technical consistency were evaluated using Pearson correlation analysis, principal component analysis (PCA), and comparison of total protein intensity distributions across samples. Spectronaut-reported normalized protein intensities were used for downstream analyses. Protein intensities were center-normalized across samples, and missing values were imputed using the K-nearest neighbors (KNN) algorithm. Reproducibility was evaluated using Pearson correlation coefficients, principal component analysis, and relative standard deviation. All samples were processed and analyzed in a single analytical batch using identical LC–MS settings.

S2 Metabolomic Analysis

S2.1 Plasma Sample Preparation

Plasma metabolomics was performed by a third-party service provider (Majorbio, Shanghai, China). For metabolite extraction, 100 uL plasma was mixed with 800 uL acetonitrile: methanol (1:1, v/v) containing internal standards (e.g., 0.02 mg/mL L-2-chlorophenylalanine), vortexed for 30 s, and sonicated at 5C (40 kHz) for 30 min. Samples were incubated at -20C for 30 min and centrifuged (13,000 g, 4C, 15 min). The supernatant was collected, dried under nitrogen, reconstituted in 100 uL acetonitrile: water (1:1, v/v), sonicated for 5 min (5C, 40 kHz), and centrifuged (13,000 g, 4C, 10 min). The final supernatant was transferred to autosampler vials for LC–MS/MS analysis.

S2.2 Quality Control (QC)

QC samples were prepared by pooling equal aliquots of all study samples and processed identically to analytical samples. QC injections were included at regular intervals to monitor instrument stability and analytical reproducibility.

S2.3 LC–MS/MS Analysis

S2.3.1 Liquid Chromatography

LC–MS/MS was performed on a UHPLC–Q Exactive HF-X system (Thermo Fisher Scientific) using an ACQUITY UPLC HSS T3 column (100 mm x 2.1 mm i.d., 1.8 um; Waters). The column temperature was 40C, flow rate 0.40 mL/min, and injection volume 3 uL. Mobile phase A was water/acetonitrile (95:5, v/v) with 0.1% formic acid, and mobile phase B was acetonitrile/isopropanol/water (47.5:47.5:5, v/v/v) with 0.1% formic acid. The LC gradient followed the vendor acquisition method.

S2.3.2 Mass Spectrometry and DDA Acquisition

Data were acquired in positive and negative electrospray ionization modes using data-dependent acquisition (DDA) over an m/z range of 70–1050. Source parameters were: source temperature 425C; sheath gas 50 arb; auxiliary gas 13 arb; spray voltage 3500

V (positive) and -3500 V (negative); and stepped normalized collision energy 20/40/60 for MS/MS.

S2.4 Raw Data Processing and Metabolite Annotation

Raw LC–MS data were processed in Progenesis QI (Waters) for peak detection, retention-time alignment, and peak area extraction, and the feature intensity matrix was exported for downstream analyses. Metabolites were annotated by matching accurate mass (MS1) and MS/MS spectra against HMDB, METLIN, and the Majorbio self-compiled database (MJDB), using an MS1 mass tolerance of 10 ppm. Identifications are reported as putative annotations because authentic reference standards were not analyzed under identical LC conditions; therefore, MSI level 1 identifications were not claimed.

S2.5 Secondary Data Processing and Quality Filtering

The feature intensity matrix and metabolite annotations (including MJDB-based annotations) were subjected to secondary data processing and statistical analyses in R (v4.3). Features were retained if they had non-zero intensities in at least 80% of all samples. Missing values were imputed using the minimum observed value. To reduce technical variability, the sample-wise sum normalization (scaling each sample to a constant total signal) was applied, followed by logarithmic transformation. Feature reproducibility was evaluated using pooled QC injections; features with QC relative standard deviation (RSD) > 30% were removed. The resulting matrix was used for downstream analyses.

Supplementary Tables

Table S1. Top 30 differentially expressed proteins between PAD patients with and without MACE.

Protein name	logFC	Adjust P
MMP3	1.14	7.96×10^{6}
ADAM15	0.82	4.38×10^{5}
MMP19	0.82	8.50×10^{5}
CCL14	0.64	0.00013
NID2	0.52	0.00013
TNFRSF6B	1.16	0.00014
CHGB	1.09	0.00029
AEBP1	0.55	0.00049
PRB2	1.03	0.00049
GM2A	1.00	0.00060
CSRP2	0.81	0.00060
SPON2	0.73	0.00060
TCEA2	0.75	0.00060
CDH11	0.69	0.00087
MT1H	0.65	0.00141
MSLN	0.56	0.00148
CBR1	0.52	0.00155
BAX	0.32	0.00155
INHBB	0.83	0.00156
CTHRC1	0.55	0.00157
MT2A	1.39	0.00175
DSC2	0.56	0.00175
B3GAT3	0.51	0.00176
ABHD14B	0.45	0.00181
IGFBP2	0.82	0.00181
IGFBP6	0.92	0.00182
MFAP5	0.42	0.00192
CRIP1	0.56	0.00192
LY96	0.64	0.00225
MYL2	0.69	0.00232

Table S2. Top 30 differentially expressed metabolomic between PAD patients with and without MACE.

Metabolism name	logFC	adjustP
Indolepyruvate	1.11	2.82×105
8-Amino-7-oxononanoic acid	1.01	2.82×105
p-Anisic acid	0.63	2.82×105
gamma-Aminobutyric acid	0.36	4.33×105
Creatinine	0.76	0.00011
3-Isopropylmalic acid	0.72	0.00011
5-Hydroxy-L-tryptophan	2.02	0.00015
4-Aminohippuric acid	1.40	0.00028
O-Acetylserine	0.63	0.00032
Pseudouridine	0.59	0.00044
L-Aspartic acid	0.74	0.00057
Kynurenic acid	0.71	0.00067
Mimosine	1.37	0.00084
Dihydrothymine	1.02	0.00084
2-(Methylamino)benzoic acid	0.86	0.00084
Hydantoin-5-propionic acid	2.54	0.00084
5-Hydroxyindoleacetic acid	0.42	0.00109
D-Kynurenine	0.55	0.00109
Pimelic acid	0.59	0.00109
N-Acetyl-L-phenylalanine	0.76	0.00109
Valdecoxib	-1.35	0.00136
Oxybenzone	-1.50	0.00160
L-Homoserine	0.76	0.00160
(3R,4R,5R)-1,3,4,5,6-Pentahydroxyhexan-2-one	-1.07	0.00160
Nicotinuric acid	2.14	0.00162
Testosterone enanthate	3.99	0.001662
Gulonic acid	0.82	0.00179
Phenylacetylglutamine	1.24	0.00204
Metipranolol	0.75	0.00271
Ketoleucine	-0.47	0.00279
Salicylhydroxamic acid	1.53	0.00279
Kynurenic acid	0.71	0.00279
Mimosine	1.37	0.00279

Table S3. Sensitivity analysis results for the top 30 differentially expressed proteins after adjustment for pre-admission antithrombotic medication use

Protein name	logFC	Adjust P
MMP3	1.14	9.06×10^{6}
ADAM15	0.82	5.07×10^{5}
MMP19	0.81	9.85×10^{5}
CCL14	0.64	0.00015
NID2	0.52	0.00015
TNFRSF6B	1.16	0.00016
CHGB	1.09	0.00031
AEBP1	0.56	0.00043
PRB2	1.03	0.00043
CSRP2	0.81	0.00068
GM2A	1.00	0.00068
SPON2	0.73	0.00068
TCEA2	0.74	0.00068
CDH11	0.69	0.00100
MT1H	0.65	0.00148
MSLN	0.56	0.00157
BAX	0.32	0.00157
CBR1	0.52	0.00163
INHBB	0.83	0.00171
CTHRC1	0.55	0.00172
MT2A	1.39	0.00188
ABHD14B	0.45	0.00188
DSC2	0.56	0.00188
B3GAT3	0.51	0.00191
IGFBP6	0.92	0.00195
IGFBP2	0.82	0.00195
CRIP1	0.56	0.00206
MFAP5	0.42	0.00208
CST3	0.68	0.00238
LY96	0.64	0.00242

Table S4. Sensitivity analysis results for the top 30 differentially expressed proteins after adjustment for major baseline comorbidities

Protein name	logFC	Adjust P
MMP3	1.09	0.00018
ADAM15	0.70	0.0040
NID2	0.48	0.0056
MMP19	0.66	0.0056
CHGB	0.99	0.0056
CCL14	0.54	0.0061
TCEA2	0.71	0.0092
PRB2	0.93	0.0092
AEBP1	0.49	0.0097
DSC2	0.57	0.0097
GM2A	0.91	0.0098
SPON2	0.67	0.0121
TNFRSF6B	0.93	0.0142
PRG3	0.89	0.0148
CDH11	0.62	0.0149
MT1H	0.60	0.0149
B3GAT3	0.49	0.0160
NAXE	0.50	0.0192
INHBB	0.71	0.0217
LOXL2	0.66	0.0217
ASGR2	0.57	0.0247
CD5L	0.62	0.0247
XCL1	0.43	0.0247
MSLN	0.46	0.0247
VCAM1	0.48	0.0247
IGFBP2	0.72	0.0247
CBR3	0.48	0.0249
TFPI2	0.66	0.0249
FAM114A1	0.49	0.0264
SFT2D3	0.40	0.0270

Table S5. Sensitivity analysis results for the top 30 differentially expressed metabolomic after adjustment for pre-admission antithrombotic medication use

Metabolism name	logFC	adjustP
Indolepyruvate	1.11	2.82×10^{5}
8-Amino-7-oxononanoic acid	1.01	2.82×10^{5}
p-Anisic acid	0.63	2.82×10^{5}
gamma-Aminobutyric acid	0.36	4.33×10^{5}
Creatinine	0.76	0.00011
3-Isopropylmalic acid	0.72	0.00011
5-Hydroxy-L-tryptophan	2.02	0.00015
4-Aminohippuric acid	1.40	0.00028
O-Acetylserine	0.63	0.00032
Pseudouridine	0.59	0.00044
L-Aspartic acid	0.74	0.00057
Kynurenic acid	0.71	0.00067
Mimosine	1.37	0.00084
Dihydrothymine	1.02	0.00084
2-(Methylamino)benzoic acid	0.86	0.00084
Hydantoin-5-propionic acid	2.54	0.00084
5-Hydroxyindoleacetic acid	0.42	0.00109
D-Kynurenine	0.55	0.00109
Pimelic acid	0.59	0.00109
N-Acetyl-L-phenylalanine	0.76	0.00109
Valdecoxib	-1.35	0.00136
Oxybenzone	-1.50	0.00160
L-Homoserine	0.76	0.00160
(3R,4R,5R)-1,3,4,5,6-Pentahydroxyhexan-2-one	-1.07	0.00160
Nicotinuric acid	2.14	0.00162
Testosterone enanthate	3.99	0.001662
Gulonic acid	0.82	0.00179
Phenylacetylglutamine	1.24	0.00204
Metipranolol	0.75	0.00271
Ketoleucine	-0.47	0.00279
Salicylhydroxamic acid	1.53	0.00279
Kynurenic acid	0.71	0.00279
Mimosine	1.37	0.00279

Table S6. Sensitivity analysis results for the top 30 differentially expressed metabolomic after adjustment for major baseline comorbidities

Metabolism name	logFC	adjustP
Indolepyruvate	0.81	0.0088
p-Anisic acid	0.50	0.0088
8-Amino-7-oxononanoic acid	0.68	0.0090
gamma-Aminobutyric acid	0.24	0.0146
3-Isopropylmalic acid	0.55	0.0187
Creatinine	0.50	0.0213
5-Hydroxy-L-tryptophan	1.42	0.0426
PC (16:0/20:5(5Z,8Z,11Z,14Z,17Z))	-0.52	0.0461
4-Aminohippuric acid	0.92	0.0521
Hydantoin-5-propionic acid	1.96	0.0521
Pseudouridine	0.42	0.0521
2-(Methylamino)benzoic acid	0.64	0.0521
(3R,4R,5R)-1,3,4,5,6-Pentahydroxyhexan-2-one	-0.88	0.0521
Mimosine	0.96	0.0638
Kynurenic acid	0.45	0.06383
L-Aspartic acid	0.48	0.06383
Pimelic acid	0.41	0.06383
O-Acetylserine	0.38	0.06383
N-Acetyl-L-phenylalanine	0.55	0.06383
5-Hydroxyindoleacetic acid	0.31	0.06383
Valdecoxib	-0.98	0.06568
Oxybenzone	-1.14	0.06568
Scopoletin	0.91	0.06681
D-Kynurenine	0.39	0.06769
Nicotinuric acid	1.59	0.06769
Testosterone enanthate	3.03	0.06769
Dihydrothymine	0.65	0.06769
Metipranolol	0.57	0.07837
Buprenorphine	-0.54	0.07837
Deoxycholic acid	-1.71	0.07837

Table S7. Overview of the main algorithms used for feature selection in this study and relevant literature references

Algorithm	Description	References
LASSO	LASSO is a regression method that performs both variable selection and regularization to enhance the prediction accuracy and interpretability of the statistical model8. By penalizing the sum of absolute values of the regression coefficients (L1 penalty), LASSO forces some coefficients to exactly zero, effectively selecting a simpler model with only the most relevant features. This makes LASSO widely used in high-dimensional datasets where feature selection is important.	1 Tibshirani, R. Regression Shrinkage and Selection Via the Lasso. Journal of the Royal Statistical Society: Series B (Methodological) 58, 267-288, doi:10.1111/j.2517-6161. 1996.tb02080.x (2018).
Random Forest (Variable Importance)	Random Forest is an ensemble classification and regression method that constructs multiple decision trees from bootstrapped samples and aggregates predictions. It calculates variable importance scores based on measures such as mean decrease in Gini impurity and accuracy, enabling ranking of features by their influence on model performance. Robust to noise and overfitting, Random Forest is widely used in high-dimensional biological data for feature selection and predictive modeling. Top features are selected according to these importance scores for downstream analysis.	Díaz-Uriarte R and Alvarez de Andrés S. Gene selection and classification of microarray data using random forest. BMC bioinformatics 2006; 7: 3. 2006/01/10. DOI: 10.1186/1471-2105-7-3.

Supplementary Figures

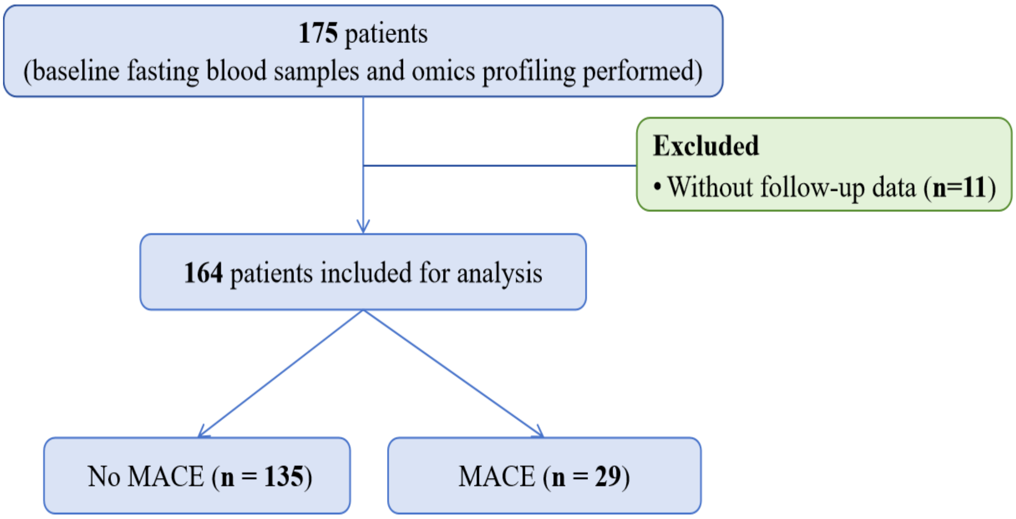

Figure S1. Patient flow diagram. Flow diagram showing recruitment and grouping of the study participants. Of 175 patients with baseline fasting blood samples and omics profiling, 11 were excluded due to missing follow-up data. A total of 164 patients were included in the final analysis, with 29 experiencing major adverse cardiovascular events (MACE) during follow-up and 135 without MACE

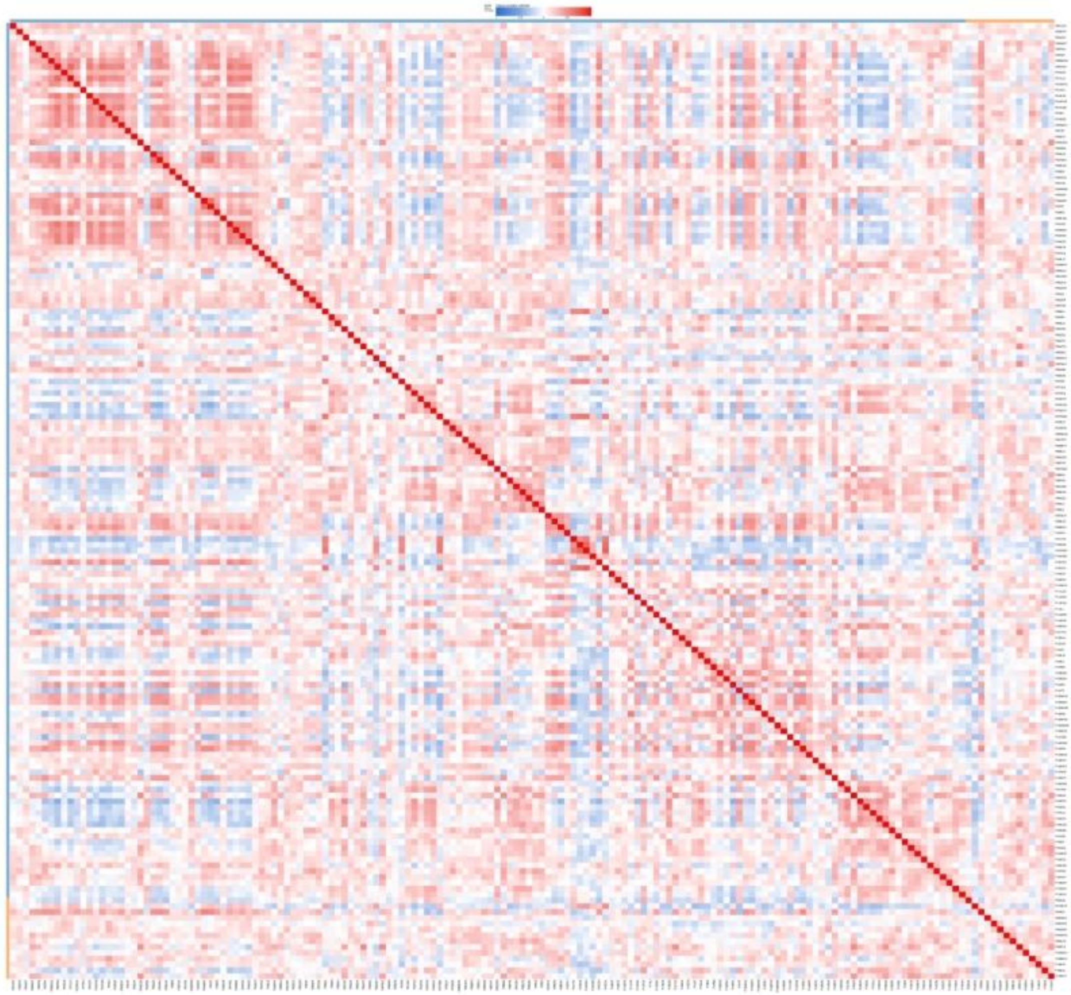

Figure S2. Pearson correlation matrix across samples. The correlation heatmap shows no clustering pattern associated with technical factors or processing order.

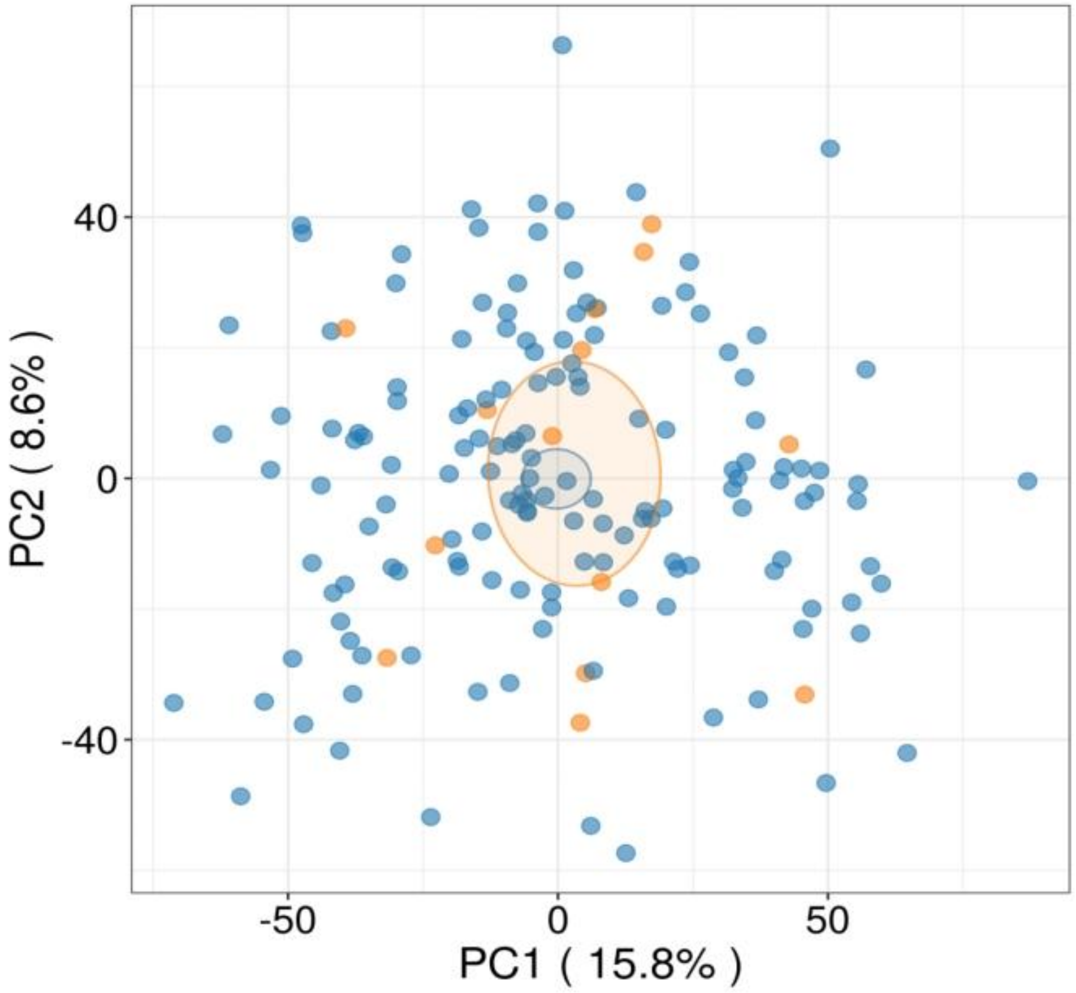

Figure S3. Principal component analysis (PCA) of proteomic profiles for all samples. PCA score plot showing the distribution of individual samples based on the first two principal components (PC1 and PC2), which explain 15.8% and 8.6% of the total variance, respectively. Each point represents a sample, colored by outcome group. The 95% confidence ellipse indicates the overall variance structure of the data. No distinct clustering by outcome group is observed, supporting the comparability of the proteomic dataset.

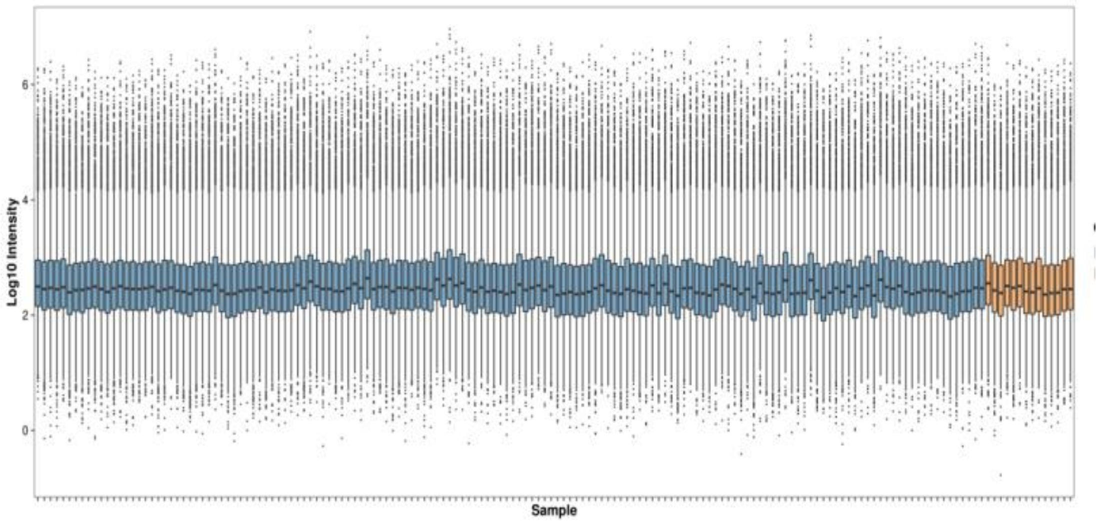

Figure S4. Distribution of proteomic signal intensities across patient groups. Boxplot of log10-transformed protein intensity values for each sample. The consistent distribution across samples indicates comparability and normalization of the proteomic data between groups.

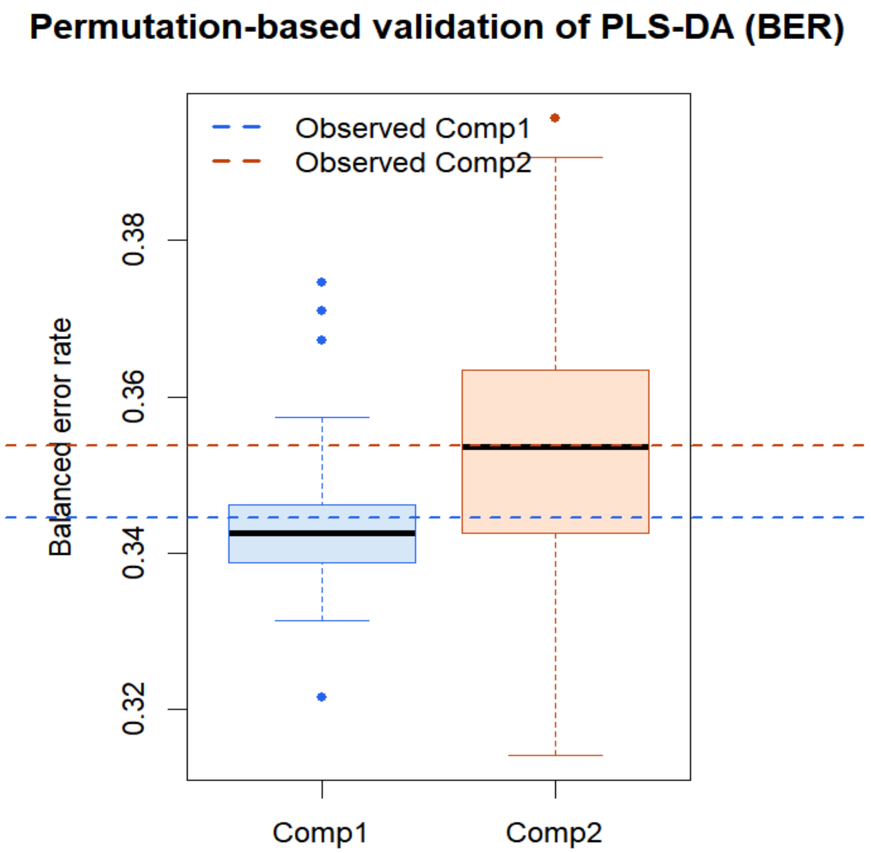

Figure S5. Permutation-based validation of PLS-DA model using balanced error rate (BER). Boxplots showing the distribution of balanced error rates (BER) for the discriminant components obtained from partial least squares discriminant analysis (PLS-DA), following permutation testing. Dashed lines indicate the observed BER for each component. Lower BER values reflect better classification performance and model robustness.

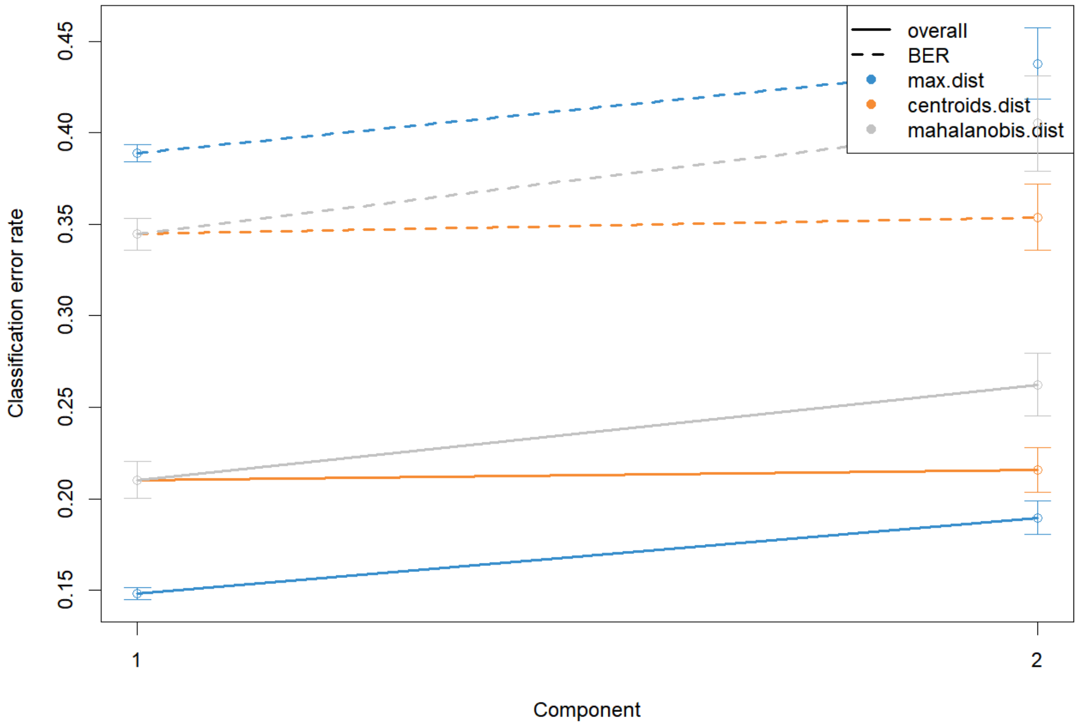

Figure S6. Classification error rates for PLS-DA components. Line plot of classification error rates for the first two PLS-DA components, assessed by overall error, balanced error rate (BER), and three distance metrics: maximum distance (max.dist), centroid distance (centroids.dist), and Mahalanobis distance (mahalanobis.dist). Error bars represent the standard error of the mean. Comparative performance across components and metrics allows evaluation of model discriminative ability.

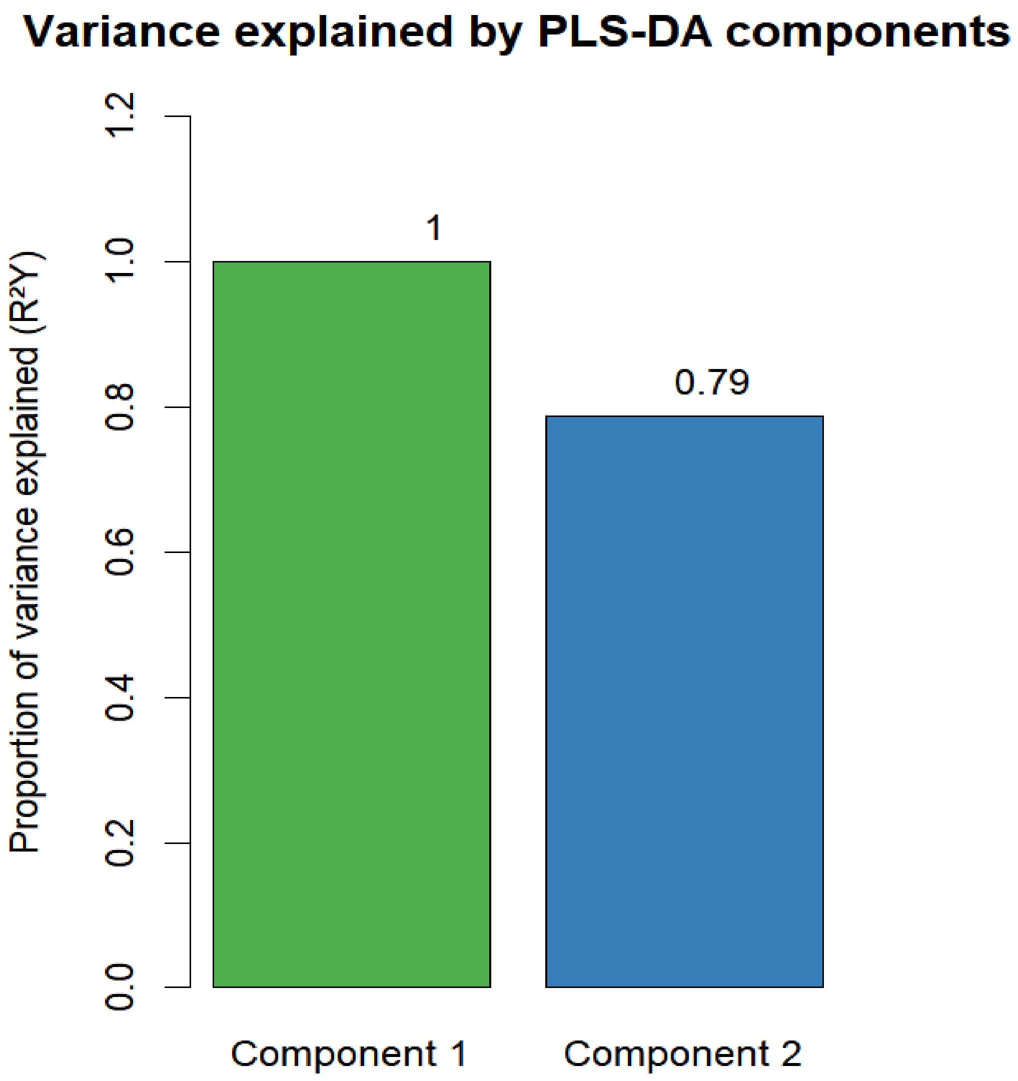

Figure S7. Variance explained by PLS-DA components. Bar plot depicting the proportion of variance explained (***R^2***) by the first two PLS-DA components. Component 1 explains the greatest amount of variance (1.00), followed by component 2 (0.79), highlighting their respective contributions to class separation in the metabolomic dataset.

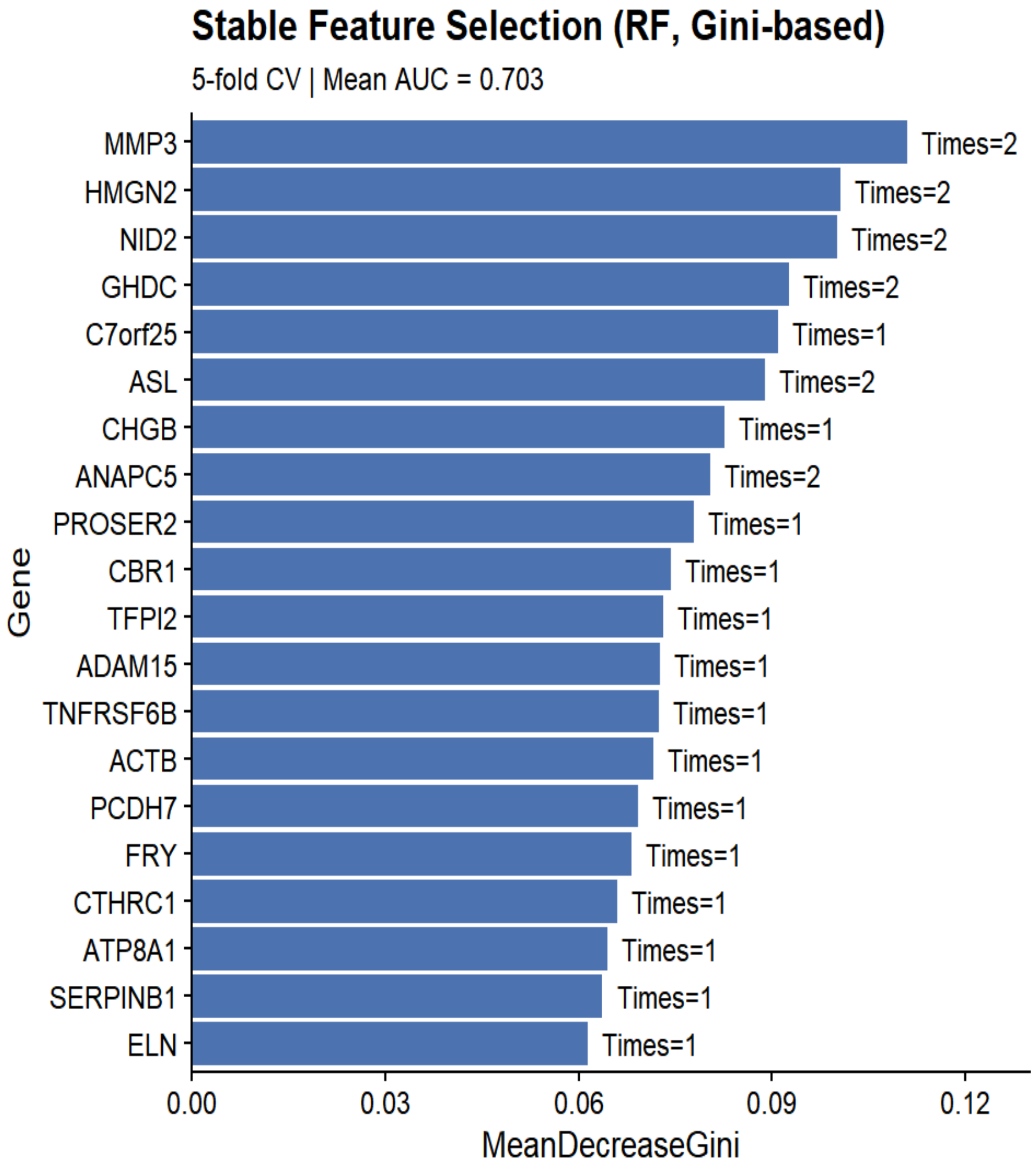

Figure S8. Stable feature selection by random forest (RF) using Gini importance. Bar plot showing the most stable protein features identified by 5-fold cross-validated random forest analysis, ranked by their mean decrease in Gini impurity. The x-axis represents the mean decrease in Gini index, reflecting variable importance, while the y-axis lists the corresponding gene symbols. The number of times each protein was selected across the cross-validation folds is indicated to the right of each bar. The mean area under the ROC curve (AUC) across all folds is 0.703.

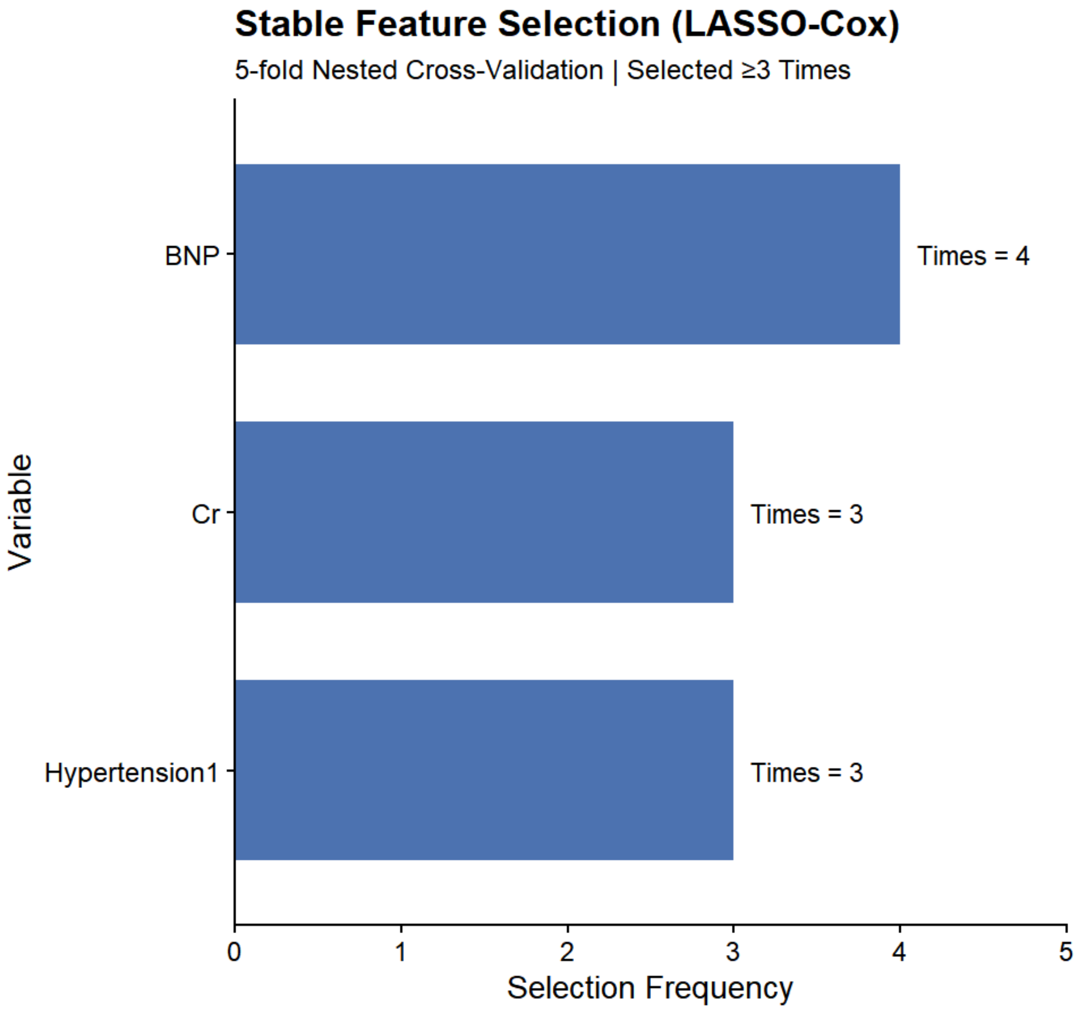

Figure S9. Stability of clinical variable selection by LASSO-Cox regression. Bar plot showing the selection frequency of clinical variable across 5-fold nested cross-validation using LASSO-Cox regression. Variables selected in at least three out of five folds were considered robust predictors for model construction.

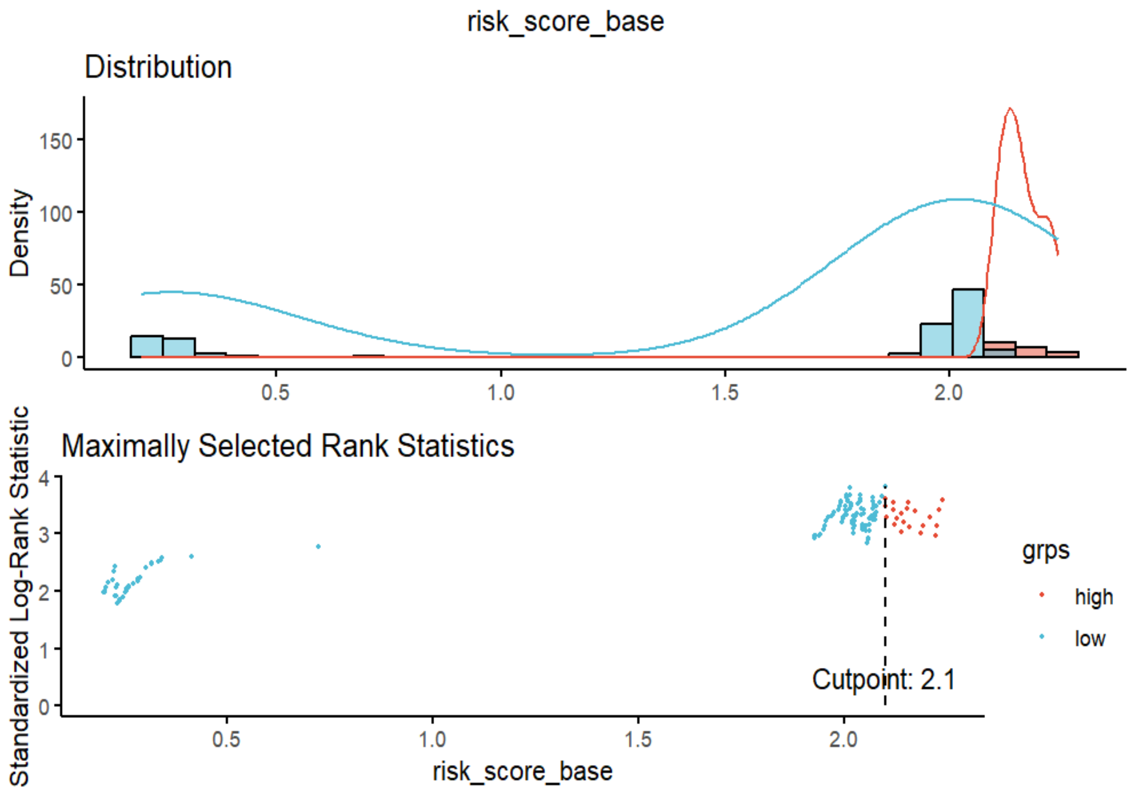

Figure S10. Determination of the optimal cutoff for clinical risk score stratification. (Top) Density plot depicting the distribution of clinical risk scores and corresponding group assignment (high vs. low risk) for individual samples. (Bottom) Maximally selected rank statistics plot for the clinical risk score, with the optimal cutoff (2.1) indicated by the dashed line. This cutoff maximizes the difference in survival between high- and low-risk groups.

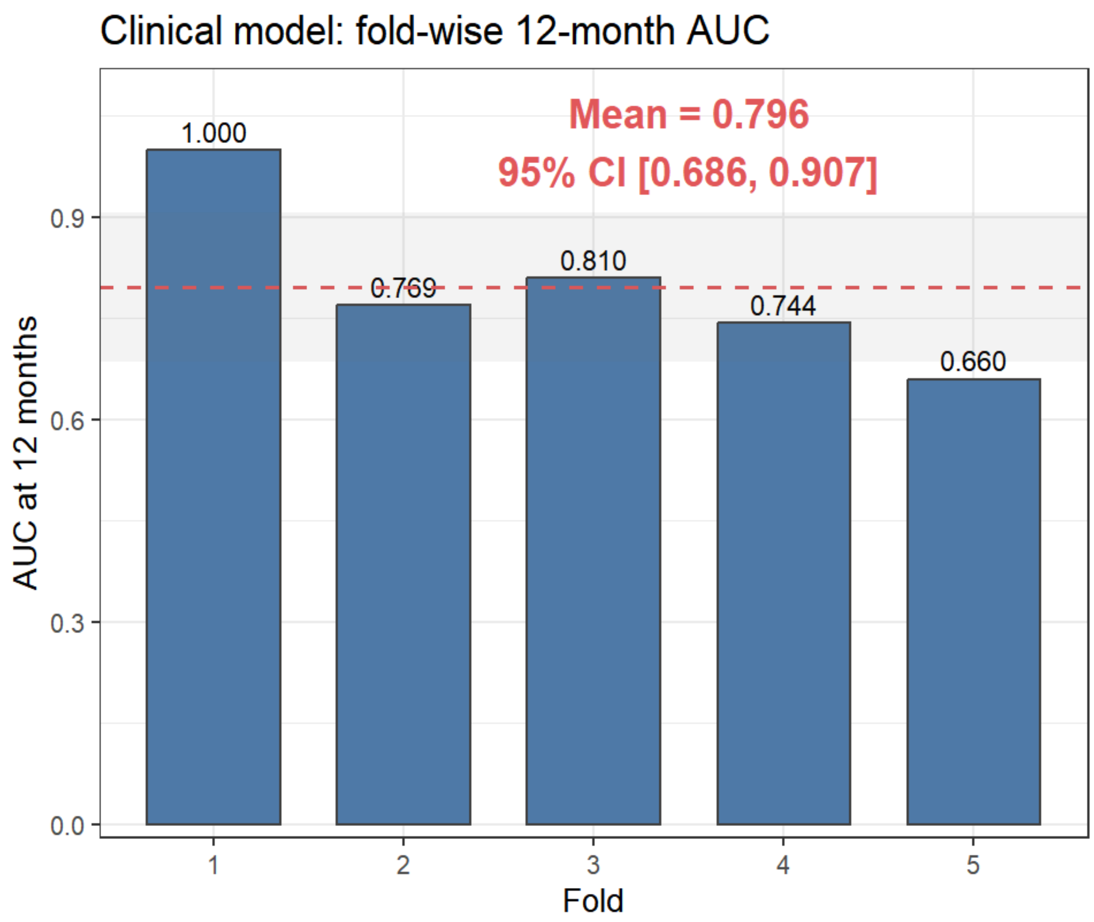

Figure S11. Fold-wise 12-month AUCs for the clinical model. Bar plot displaying the 12-month area under the curve (AUC) for each fold of the 5-fold cross-validation using the clinical model. The mean AUC (0.796) and 95% confidence interval ([0.686, 0.907]) are indicated by the red dashed line.

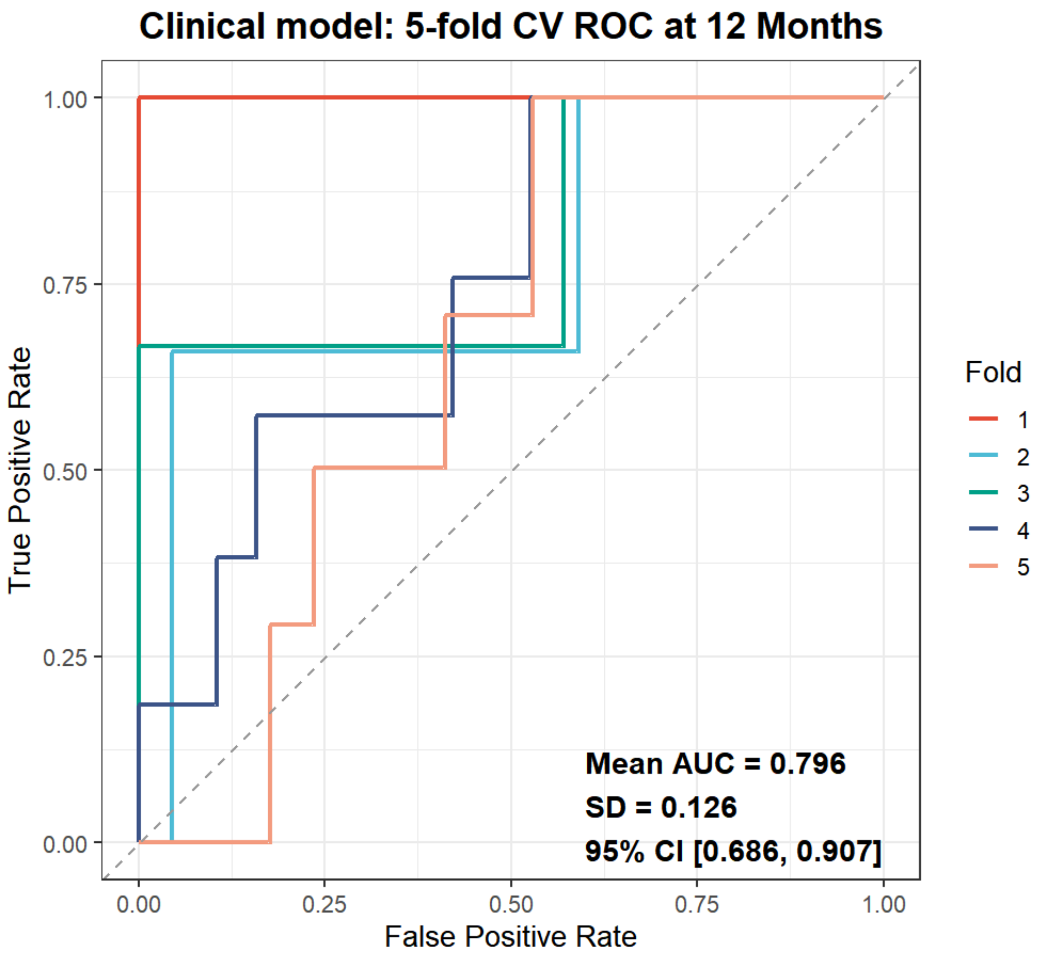

Figure S12. Fold-wise ROC curves for the clinical model at 12 months. Receiver operating characteristic (ROC) curves for each fold of 5-fold cross-validation using the clinical model. The plot demonstrates the true positive versus false positive rates for each fold, with the mean AUC, standard deviation (SD = 0.126), and 95% confidence interval indicated in the lower right.

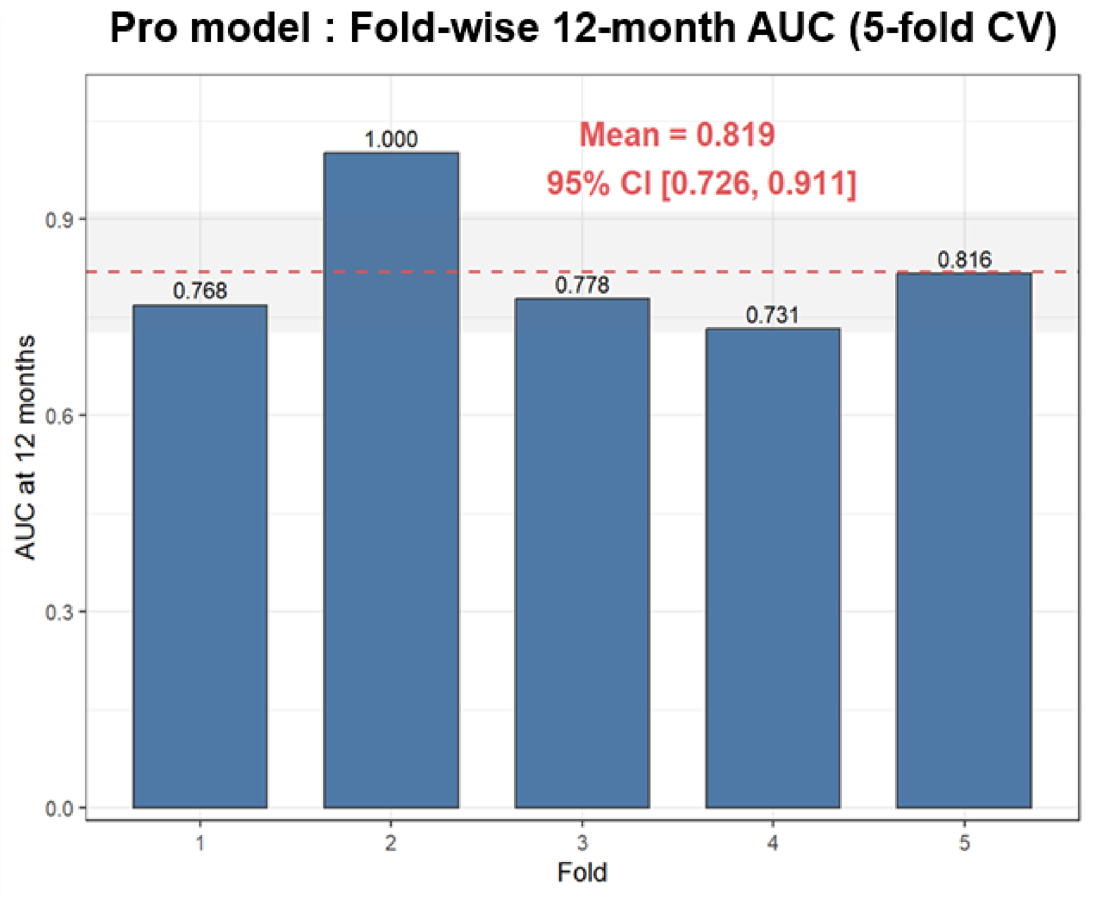

Figure S13. Fold-wise 12-month AUCs for the protein-based model. Bar plot illustrating the 12-month area under the curve (AUC) for each fold of the 5-fold cross-validation using the protein-based model. The mean AUC (0.819) and 95% confidence interval ([0.726, 0.911]) are indicated by the red dashed line.

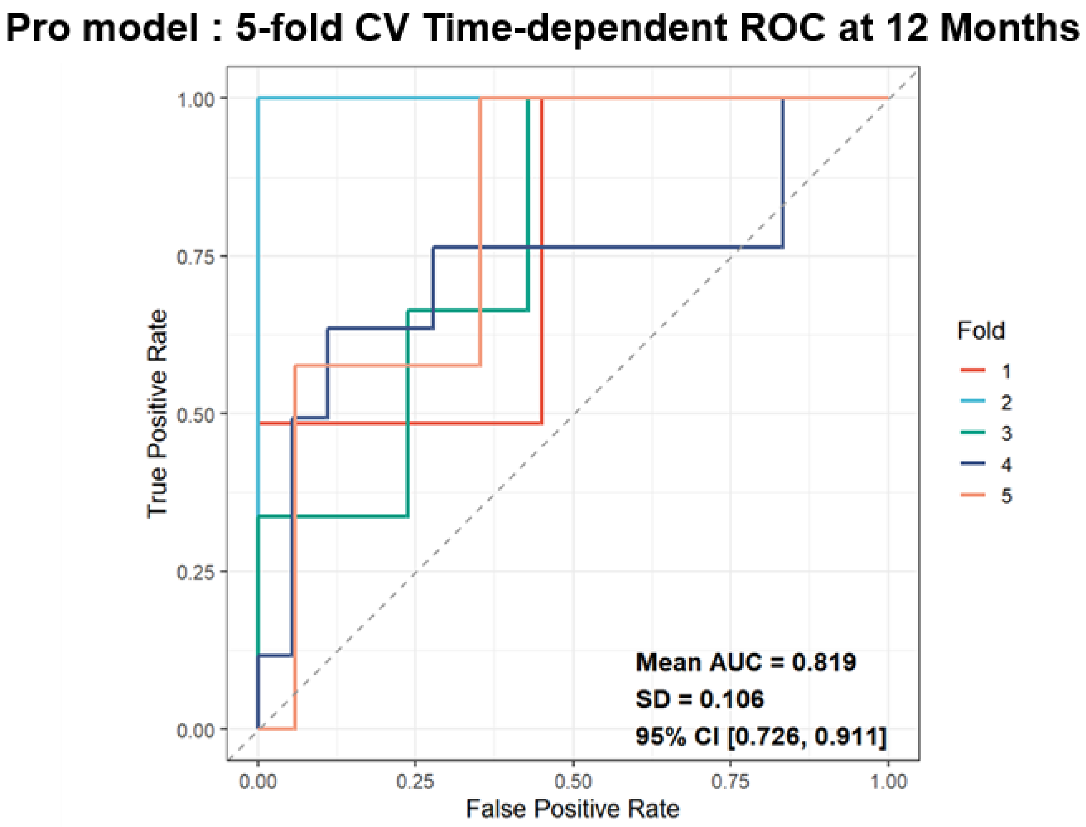

Figure S14. Fold-wise ROC curves for the protein-based model at 12 months. Receiver operating characteristic (ROC) curves for each fold of 5-fold cross-validation using the protein-based model. The plot displays the trade-off between true positive rate and false positive rate for each fold, with the mean AUC, standard deviation (SD = 0.106), and 95% confidence interval [0.726, 0.911] reported in the lower right corner.

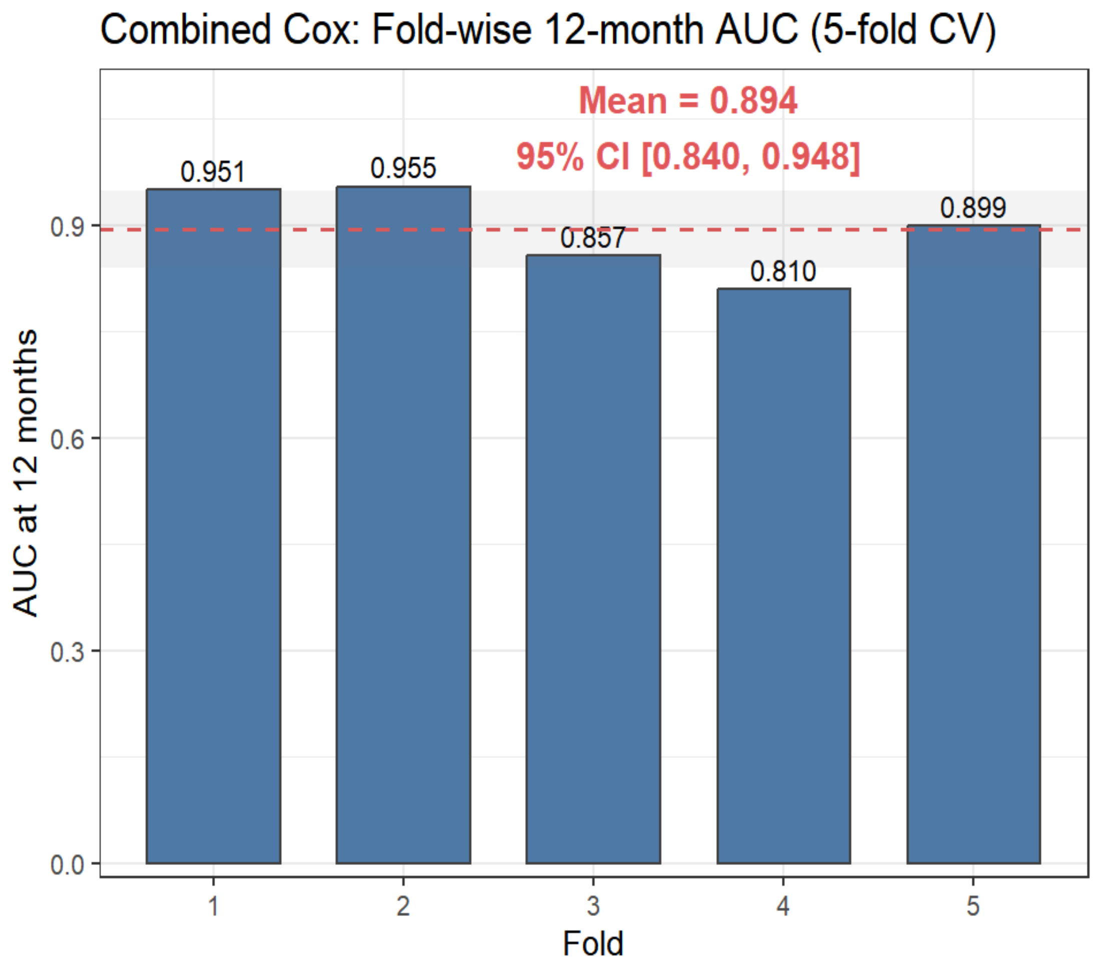

Figure S15. Fold-wise 12-month AUCs for the combined clinical and proteomic model. Bar plot presenting the 12-month area under the curve (AUC) for each fold of the 5-fold cross-validation using the combined clinical and proteomic model. The mean AUC (0.894) and 95% confidence interval [0.840, 0.948] are indicated by the red dashed line.

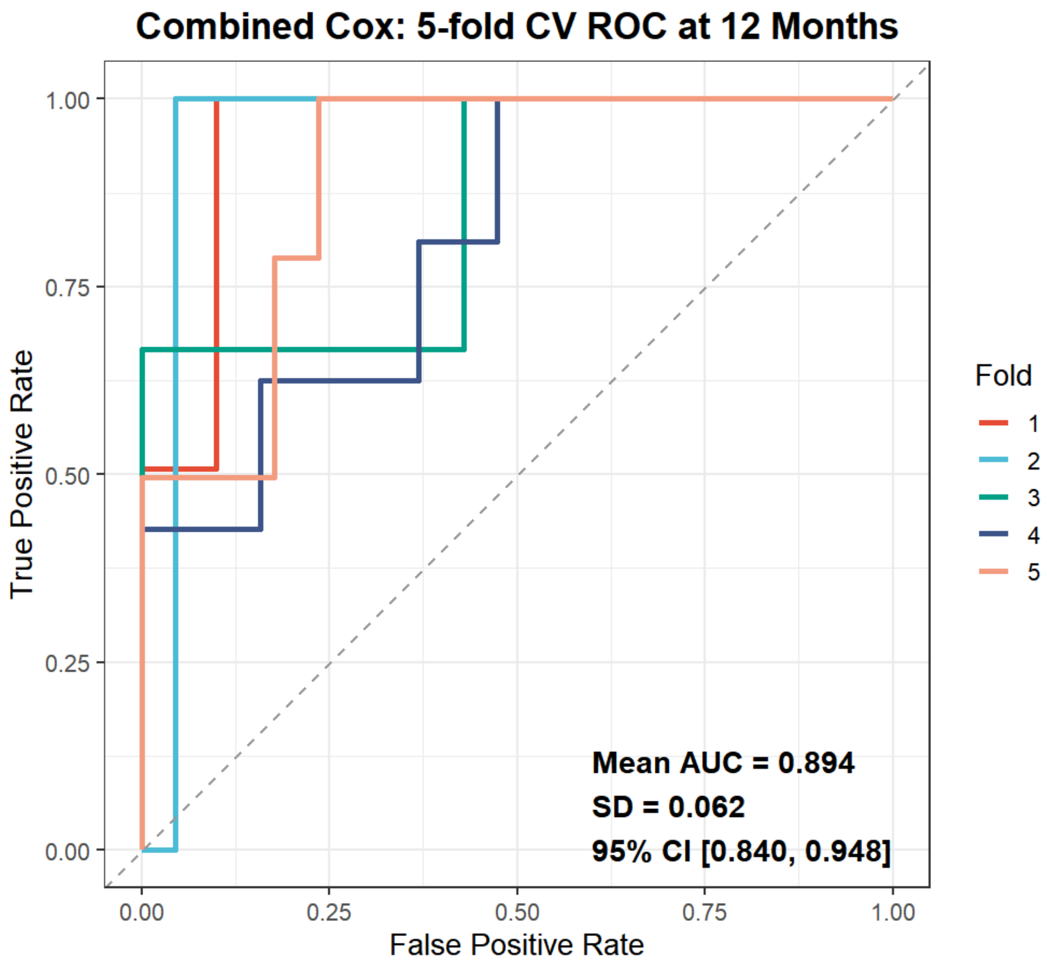

Figure S16. Fold-wise ROC curves for the combined clinical and proteomic model at 12 months. Receiver operating characteristic (ROC) curves for each fold of 5-fold cross-validation using the combined clinical and proteomic model. The mean AUC, standard deviation (SD = 0.062), and 95% confidence interval [0.840, 0.948] are shown in the lower right, demonstrating robust model performance at the 12-month landmark.
