# Supplementary figures and images for: Prediction of Major Adverse Cardiovascular Events in Peripheral Artery Disease: Integrating Metabolomics and Proteomics for Risk Stratification
Source: Research (Wash D C). 2026 May 6;9:1229. doi: 10.34133/research.1229 (PMC13148185; doi:10.34133/research.1229)

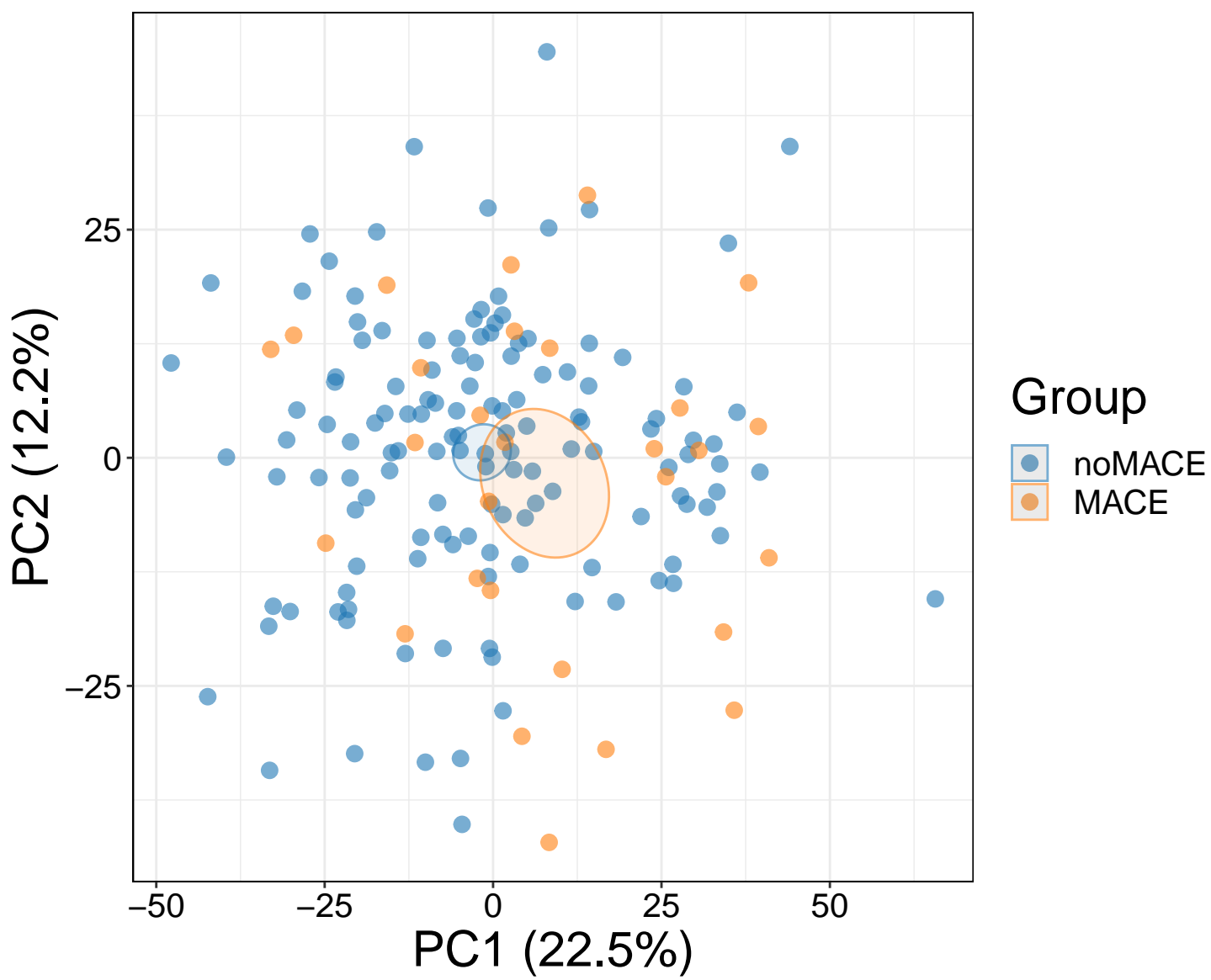

Supplement: Supplementary 1 — Supplementary Methods Tables S1 to S7 Figs. S1 to S16 Data S1 to S10 [file research.1229.f1.zip › Figure S3.pdf]

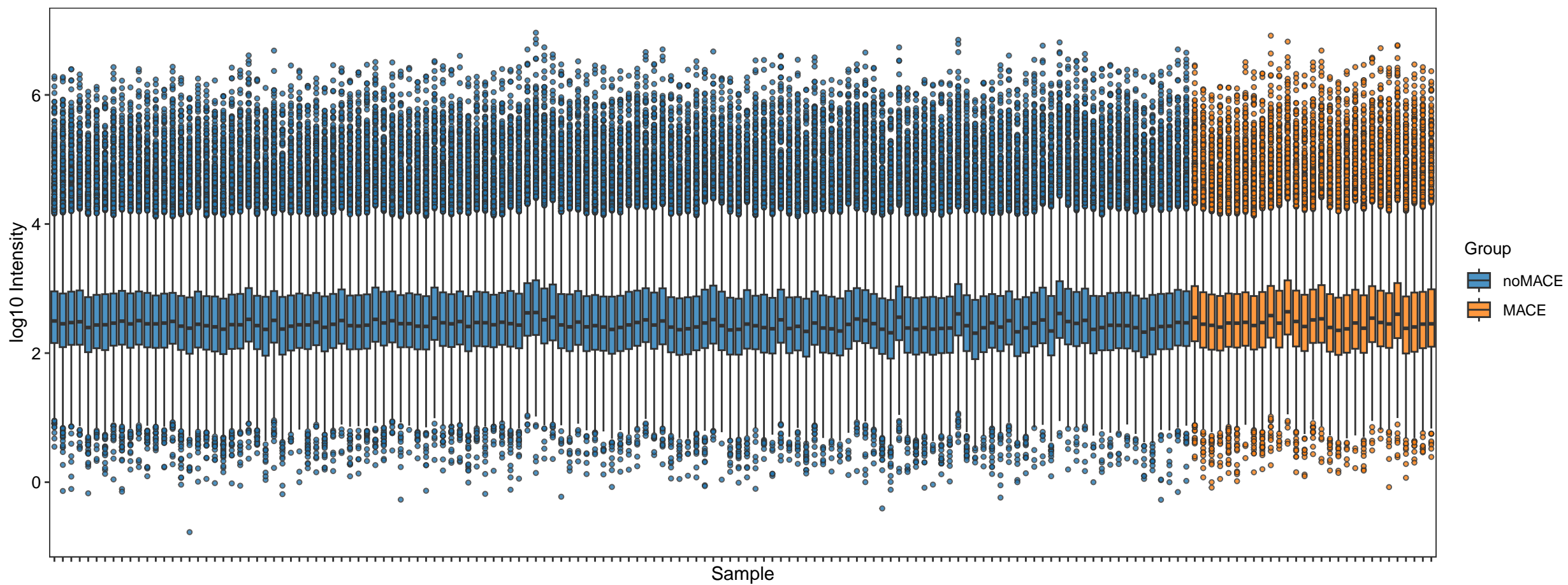

Supplement: Supplementary 1 — Supplementary Methods Tables S1 to S7 Figs. S1 to S16 Data S1 to S10 [file research.1229.f1.zip › Figure S4.pdf]
